# The complete mitochondrial genome of *Castanopsis carlesii* and *Castanea henryi* reveals the rearrangement and size differences of mitochondrial DNA molecules

**DOI:** 10.1186/s12870-024-05618-z

**Published:** 2024-10-21

**Authors:** Xiong-De Tu, Ya-Xuan Xin, Hou-Hua Fu, Cheng-Yuan Zhou, Qing-Long Liu, Xing-Hao Tang, Long-Hai Zou, Zhong-Jian Liu, Shi-Pin Chen, Wen-Jun Lin, Ming-He Li

**Affiliations:** 1 College of Forestry, Fujian Agriculture and Forestry University, Fuzhou, 350002 China; 2 Key Laboratory of National Forestry and Grassland Administration for Orchid Conservation and Utilization at College of Landscape Architecture, College of Landscape Architecture, Fujian Agriculture and Forestry University, Fuzhou, 350002 China; 3 State Key Laboratory of Subtropical Silviculture, Bamboo Industry Institute, Zhejiang A&F University, Lin’an, Hangzhou, 311300 China

**Keywords:** *Castanopsiscarlesii*, *Castaneahenryi*, Homologous recombination, Mitogenome, Fagaceae

## Abstract

**Background:**

*Castanopsis carlesii* is a dominant tree species in subtropical evergreen broad-leaved forests and holds significant ecological value. It serves as an excellent timber tree species and raw material for cultivating edible fungi. Henry Chinquapin (*Castanea henryi*) wood is known for its hardness and resistance to water and moisture, making it an exceptional timber species. Additionally, its fruit has a sweet and fruity taste, making it a valuable food source. However, the mitogenomes of these species have not been previously reported. To gain a better understanding of them, this study successfully assembled high-quality mitogenomes of *C. carlesii* and *Ca. henryi* for the first time.

**Results:**

Our research reveals that the mitochondrial DNA (mtDNA) of *C. carlesii* exhibits a unique multi-branched conformation, while *Ca. henryi* primarily exists in the form of two independent molecules that can be further divided into three independent molecules through one pair of long repetitive sequences. The size of the mitogenomes of *C. carlesii* and *Ca. henryi* are 592,702 bp and 379,929 bp respectively, which are currently the largest and smallest Fagaceae mitogenomes recorded thus far. The primary factor influencing mitogenome size is dispersed repeats. Comparison with published mitogenomes from closely related species highlights differences in size, gene loss patterns, codon usage preferences, repetitive sequences, as well as mitochondrial plastid DNA segments (MTPTs).

**Conclusions:**

Our study enhances the understanding of mitogenome structure and evolution in Fagaceae, laying a crucial foundation for future research on cell respiration, disease resistance, and other traits in this family.

**Supplementary Information:**

The online version contains supplementary material available at 10.1186/s12870-024-05618-z.

## Background

*Castanopsis carlesii* (Hemsl.) Hayata and *Castanea henryi* (Skan) Rehder & E. H. Wilson belong to the Fagaceae, which comprises eight genera and over 900 species [1, 2]. The wood of *C. carlesii* has strength, durability, and resistance to rot, making it highly suitable for construction, furniture production, and other wood products. Additionally, *C. carlesii* has a high starch content in nuts along with rich trace elements, offering significant potential for development and utilization. Moreover, *C. carlesii* plays a crucial role in subtropical evergreen broad-leaved forests as a dominant tree species by contributing to hydrology regulation, erosion prevention, nutrient cycling, carbon storage, and biodiversity conservation [3–5]. *Ca. henryi* belongs to the genus *Castanea*, which is often referred to as "woody cereals". Historically, chestnut tree have been valuable for their edible fruits and durable wood [6]. The wood of *Ca. henryi* is durable and water-resistant, suitable for sleepers, buildings, ships, furniture, and wine barrels [7]. Chestnut cupules can be utilized for extracting tannic acid and tannin extracts which serve as essential raw materials within the leather industry [8]. Overall, *Ca. henryi* exhibits great potential across diverse fields like biomedicine, chemical wood industries, and food industries.

Mitochondria are widely believed to have originated from endophytic bacteria [9], serving as the "powerhouses" of cells by supplying eukaryotes with ATP energy [10, 11]. Organelles not only play a role in apoptosis, ion homeostasis, and intermediary metabolism but also hold significant importance in male sterility [12–15].

Plant mitogenomes vary greatly in size, from 66 kb to 11.3 Mb [16, 17], mainly due to non-coding sequences such as intracellular gene transfer or horizontal gene transfer, as well as differences in repeat sequences. Plant mtDNA structure is complex. While typically described as round, it can also be linear, branched, or consist of multiple small circular molecules [18–20]. For instance, in the Fagaceae family, the mitogenome assembly of *Quercus acutissima* reveals three branched structures consisting of one linear molecule and two circular molecules [21], whereas the *Ca. mollissima* mitogenome comprises two cyclic molecules [22], and the *Lithocarpus litseifolius* mitogenome forms a large circular genome [23]. These examples highlight the diverse forms of mtDNA structures observed in Fagaceae. Mitogenomes often undergo intra- or intermolecular genetic recombination through long repetitive sequences, resulting in molecular subunit genomes or isoforms [24, 25]. Restoring plant mtDNA conformation is challenging due to redundant sequences and extensive genome reorganization. With advancements in third-generation sequencing technology and assembly strategies, it is increasingly feasible to reveal intricate conformation of complex mitogenomes.

In this study, we used Illumina short reads and PacBio long reads to successfully assemble high-quality mitogenomes of *C. carlesii* and *Ca. henryi*. The objective of this study was to analyze the mitogenome recombination mediated by repetitive sequences in *C. carlesii* and *Ca. henryi*, compare the relative synonymous codon usage (RSCU), RNA editing sites and segments transfer between chloroplasts and mitogenomes in the mitochondrial genomes of seven Fagaceae species, as well as construct phylogenetic relationships of Fagaceae and related taxa based on mitochondrial genomics. The findings are expected to provide a theoretical foundation for species identification, biological research, and breeding within the Fagaceae.

## Material and methods

### Plant material, DNA extraction, and sequencing

The plant materials were obtained from wild *Castanopsis carlesii* grown in Gushan Mountain, Fuzhou City, China (26° 05′ N, 119° 39′ E) and wild *Castanea henryi* grown in Nanping City, China (27° 02′ N, 118° 44′ E). The identification of plants was conducted by Shi-Pin Chen. The specimens of *C. carlesii* and *Ca. henryi* are deposited in the Herbarium of College of Forestry, Fujian Agriculture and Forestry University (FJFC), with voucher numbers Cas019(FJFC) and Cas020(FJFC), respectively. Total DNA was extracted from fresh leaves using a modified CTAB method as described by Doyle [26]. The Illumina protocol was used to construct 500 bp paired-end libraries. We performed 20-kb single-molecule real-time (SMRT) DNA sequencing on the PacBio sequel platform.

### Assembly and annotation

The raw short reads and long reads were polished using Trimmomatic v0.32 [27] and Canu v2.2 [28], respectively, to obtain high-quality clean reads. The polished long reads were assembled using PMAT v1.2.2 [29] with the autoMito parameters. PMAT processes polished long reads by segmenting them into shorter fragments with varying step lengths (default: 20 kb) using ‘break_long_reads.py’, and subsequently assembles these fragments using the Newbler assembly software. To identify potential seed contigs, PMAT performs a BLASTn [30] search against a local database of 24 conserved plant mitochondrial protein-coding genes (PCGs) using the ‘PMATAllContigs.fna’ file. The results are then filtered through ‘find_candidate_seeds.py’ to select suitable seed contigs for extension. Next, PMAT employs ‘seeds_extension.py’ to extend the seed contigs and recruit all target mitochondrial contigs based on their connections outlined in the ‘PMATContigGraph.txt’ file. The identified contigs and their connections are subsequently inputted into ‘assembly_graph.py’ to generate the initial assembly graph (PAMA_raw.gfa) utilizing information from the ‘PMATAllContigs.fna’ file. PMAT utilizes ‘assembly_graph.py’ to eliminate non-mitochondrial contigs, such as chloroplast or nuclear contigs, from the initial mitogenome assembly graph. Following the assumption that mitogenome topology should be represented as a single circular or linear molecule, PMAT further removes certain unconnected contigs from the assembly graph that do not exhibit self-connections as circles nor any connections with other contigs, resulting in a simplified assembly graph (PAMA_master.gfa). The simplified assembly graph files generated by the PMAT software were visualized using Bandage v0.8.1 [31]. Due to the low accuracy of PacBio long readings, short reads were used twice to calibrate the assembly results based on Pilon v1.24 [32]. The mitochondrial genome was annotated using the online tool IPMGA (http://www.1kmpg.cn/ipmga/) and manually adjusted by Geneious v11.1.5 [33]. To ensure clarity in annotation, we re-annotated the mitogenome obtained from GenBank using the above method. The final visualization of the mitochondrial map was performed using OGDRAW v1.3.1 [34].

### Homologous recombination of mediated by repeats

To determine whether the three pairs of repeat sequences in the mitogenome of *C. carlesii* and *Ca. henryi* are involved in recombination, we extracted sequence fragments that included both the repeat sequences and sequences with a length of more than 5000 bp on either side of the repeat. We initially mapped long reads to each potential recombinant sequence using BWA-mem [35]. Then, we calculated the number of mapped reads using samtools v1.3.1 [36] and visualized the results using IGV v2.17.4 [37]. For a mapping to be considered valid, it had to cover at least 50 bp of flanking sequence on each side of the repeat unit. The average number of mappings obtained from two different paths for the same repetitive sequence was used as its mapping count (except for LR9 in *Ca. henryi* where direct calculation was possible due to complete coverage by long reads). Finally, we utilized the main conformations for annotation and subsequent analysis.

### Repeat sequence, Codon usage and prediction of RNA editing sites analysis

The simple sequence repeats (SSRs) were determined using the MISA web server [38] with thresholds of 10, 5, 4, 3, 3, and 3 for mono-, di-, tri-, tetra-, penta-, and hexan-repeat sequences respectively. Tandem repeats were analyzed using the Tandem Repeats Finder web service [39] with default parameters. The length and location of dispersed repeats were detected by the REPuter web server [40] with a Hamming distance of 3, minimal repeat size of 30 bp, and maximum computed repeats set to 5000.

Codon usage and RSCU values were calculated for unique PCGs using CodonW software [41]. The RNA editing sites in the mitogenome were predicted using Deepred-Mt software [42] with a probability threshold set to be at least or equal to 0.9.

### Identification of mitochondrial plastid DNA segments and synteny analysis

The chloroplast genomes of *C. carlesii* (MK840978), *Ca. henryi* (MH998384), *Quercus variabilis* (MK105451), and *Fagus sylvatica* (MK598696) were downloaded from the GenBank website (https://www.ncbi.nlm.nih.gov/). The mitogenomes were also obtained from the GenBank, and their accession numbers are listed in Table S1. We used BLASTN v2.13.0 [30] to compare the homologous sequences between the mitogenome and the chloroplast genome of each species, setting a threshold of 1e-5 for the E-value. The results were visualized using the online tool circoletto [43].

For multiple sequence alignment of mitogenomes belonging to seven Fagaceae species, AliTV v1.0.6 [44] was employed followed by visualization on a dedicated website (https://alitvteam.github.io/AliTV/d3/AliTV.html). To filter out shorter sequences, only those with a length greater than 500 bp were considered for analysis. Repeated sequences larger than 400 bp are indicated in the generated results.

#### Phylogenetic analysis

Nineteen mitogenomes from Fagales and Cucurbitales species (including seven Fagaceae species, one Juglandaceae species, ten Cucurbitaceae species, and one Begoniaceae species) were selected for phylogenetic analysis, and three Rosales species was set as the outgroup. Detailed information about each species and their accession numbers can be found in Table S1. The PCGs were extracted from mitogenomes using PhyloSuite v.1.1.16 [45]. Repetitive sequences were removed to obtain a final set of 39 protein-coding regions for constructing the phylogenetic tree. The sequences were aligned using MAFFT v7.490 [46] with default parameters. To minimize systematic errors from poor quality, we used trimAl v1.2 [47] with the ‘automated1’ parameter, a heuristic method that automatically determines the best trimming approach for aligning only high-quality regions of the 39 protein-coding genes. Finally, the trimmed 39 PCGs were concatenated to construct a phylogenetic tree using IQ-TREE v2.0.3 [48]. The IQ-TREE software combines SH-aLRT test and ultrafast bootstrap (UFBoot), with model selection performed by ModelFinder (-alrt 1000 -bb 1000 -m MFP).

## Results

### Assembly of mitochondrial genomes

**Fig. 1:**
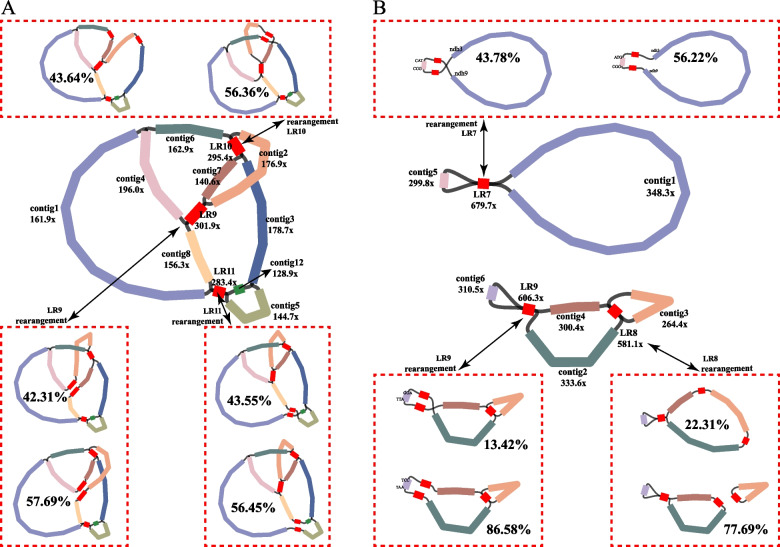
The assembly graph of the *C. carlesii* (**A**) and *Ca. henryi* (**B**) mitogenomes. Each colored fragment is labeled with its coverage depth and named in order of size. The adjacency relationships between all fragments are supported by long-read sequencing data, and the possible structures and proportion formed by rearrangements mediated by three long repeats were drew

These two species, *Castanopsis carlesii* and *Castanea henryi*, yielded 54 Gb and 63 Gb of raw reads respectively when sequenced on the Illumina platform, while the PacBio platform generated 93 Gb and 79 Gb of raw reads for each species. The resulting GFA file was then assembled using PMAT software, followed by manually deleting sequences with meager coverage or those that did not belong. The results correspond to the mitogenome structure of *C. carlesii* and *Ca. henryi* (Fig. 1), with contigs numbered based on their length (Table 1).

**Table 1:** The location, length and depth of each assembled contig in *C. carlesii* and *Ca. henryi*

Species	Sequences	Start	End	Length (bp)	Depth
*C. carlesii*	contig1	1	177,754	177,754	161.9x
	contig2	463,145	536,073	72,929	176.9x
	contig3	232,048	298,721	66,674	178.7x
	contig4	345,375	408,273	62,899	196.0x
	contig5	184,028	232,047	48,020	144.7x
	contig6	308,033	345,374	37,342	162.9x
	contig7	421,503	453,833	32,331	140.6x
	contig8	549,303	581,277	31,975	156.3x
	LR9	408,274	421,502	13,229	301.9x
		536,074	549,302		
	LR10	298,722	308,032	9,311	295.4x
		453,834	463,144		
	LR11	177,755	184,027	6,273	283.4x
		581,278	587,550		
	contig12	587,551	592,702	5,152	128.9x
*Ca. henryi*	contig1^a^	22,251	249,151	226,901	348.3x
	contig2^b^	651	55,895	55,245	333.6x
	contig3^c^	651	41,051	40,401	264.4x
	contig4^b^	65,543	86,851	21,309	300.4x
	contig5^a^	1	19,374	19,374	299.8x
	contig6^b^	56,377	65,061	8,685	310.5x
	LR7^a^	19,375	22,250	2,876	679.7x
		249,152	252,027		
	LR8^b^	1	650	650	581.1x
	LR8^c^	1	650		
	LR9^b^	55,896	56,376	481	606.3x
		65,062	65,542		

^a^Represents located in mitochondrial chromosome 1

^b^Represents located in mitochondrial chromosome 2

^c^Represents located in mitochondrial chromosome 3

The *C. carlesii* mitogenome exhibits a complex multi-branched conformation, but it remains a closed-loop structure with an average coverage depth of 164.8x. The *C. carlesii* mitogenome consists of nine contigs (contig1–8 and contig12) ranging in length from 5152 bp to 177,754 bp, along with three pairs of long repeat sequences (LR9–11) spanning from 6273 bp to 13,229 bp (Fig. 1A, Table 1). On the other hand, the *Ca. henryi* mitogenome comprises two independent molecules; one is divided into two chromosome molecules due to the LR8 repeat sequence and ultimately forms three circular chromosome molecules. The average coverage depth for mtDNA1 is 344.4x, for mtDNA2 is 322.5x, and for mtDNA3 is 264.8x. The *Ca. henryi* mitogenome includes six contigs (contig1–6) ranging in length from 8685 bp to 226,901 bp as well as three pairs of long repeat sequences (LR7–9) with lengths varying between 481 and 2876 bp (Fig. lB, Table 1).

The *C. carlesii* mitogenome contains 39 PCGs (36 unique), 22 tRNA genes (20 unique), and 4 rRNA genes (3 unique) (Table 2), while the *Ca. henryi* mitogenome contains 37 PCGs (36 unique), 22 tRNA genes (21 unique), and 3 unique rRNA genes (Table 2).

**Table 2:** The annotation results of the *C. carlesii* and *Ca. henryi* mitogenomes

Group of genes	*C. carlesii*	*Ca. henryi*
ATP synthase	*atp*1(2), *atp*4, *atp*6, *atp*8, *atp*9(2)	*atp*1, *atp*4, *atp*6, *atp*8, *atp*9(2)
Cytohrome c biogenesis	*ccm*B, *ccm*C, *ccm*FC*, *ccm*FN	*ccm*B, *ccm*C, *ccm*FC*, *ccm*FN
Ubichinol cytochrome c reductase	*cob*	*cob*
Cytochrome c oxidase	*cox*1, *cox*2, *cox*3	*cox*1, *cox*2*, *cox*3
Maturases	*mat*R	*mat*R
Transport membrance protein	*mtt*B	*mtt*B
NADH dehydrogenase	*nad*1****, *nad*2****, *nad*3, *nad*4***, *nad*4L, *nad*5****, *nad*6, *nad*7****, *nad*9	*nad*1****, *nad*2****, *nad*3, *nad*4***, *nad*4L, *nad*5****, *nad*6, *nad*7****, *nad*9
Ribosomal proteins (LSU)	*rpl*2*, *rpl*5, *rpl*10, *rpl*16	*rpl*2*, *rpl*5, *rpl*10, *rpl*16
Ribosomal proteins (SSU)	*rps*1, *rps*3*, *rps*4, *rps*10*, *rps*12, *rps*19	*rps*1, *rps*3*, *rps*4, *rps*10*, *rps*12, *rps*19
Succinate dehydrogenase	*sdh*3, *sdh*4(2)	*sdh*3, *sdh*4
Ribosomal RNAs	*rrn*5(2), *rrn*18, *rrn*26	*rrn*5, *rrn*18, *rrn*26
Transfer RNAs	*trn*C-GCA, *trn*D-GUC, *trn*E-UUC, *trn*E-UUC*(2), *trn*F-GAA, *trn*G-GCC, *trn*G-GCC*, *trn*H-GUG, *trn*I-CAU, *trn*K-UUU, *trn*M-CAU, *trn*N-GUU, *trn*P-UGG(2), *trn*Q-UUG, *trn*S-CGA, *trn*S-GCU, *trn*S-UGA, *trn*W-CCA, *trn*Y-GUA, *trnf*M-CAU	*trn*C-GCA, *trn*D-GUC(2), *trn*E-UUC, *trn*F-GAA, *trn*G-GCC, *trn*H-GUG, *trn*I-CAU, *trn*I-GAU*, *trn*K-UUU, *trn*M-CAU, *trn*N-GUU, *trn*P-UGG(2), *trn*Q-UUG, *trn*S-CGA, *trn*S-GCU, *trn*S-UGA, *trn*V-GAC, *trn*W-CCA, *trn*Y-GUA, *trnf*M-CAU

^*^Gene that contains one intron; ***Gene that contains three introns; ****Gene that contains four introns; Gene(2): Number of copies of multi-copy genes

### Homologous recombination of mitogenome mediated by repeats

We used long reads aligned to repeat sequences in different conformations and flanking them to estimate the ratio of recombinant structures. In the *C. carlesii* mitogenome, the proportions of mitogenome recombination mediated by three pairs of long repeat sequences were similar, respectively 42.31% vs. 57.69% (LR9), 43.64% vs. 56.36% (LR10), and 43.55% vs. 56.45% (LR11) (Fig. 1A, Table 3, Fig. S1–S12). In the *Ca. henryi* mitogenome, three pairs of long repeat sequences (LR7, LR8, and LR9) mediate the recombination of the mitogenome. Among them, the LR7 long repeat sequence mediates the recombination of molecule 1, and the ratio of the two isoforms is 43.78%. vs. 56.22%. Two pairs of long repeat sequences, LR8 and LR9, mediate the recombination of molecule 2, and the proportions of isoforms they mediate are 13.42% vs. 86.58% and 22.31% vs. 77.69%, respectively (Fig. 1B, Table 3, Fig. S13–S21).

**Table 3:** The number and proportion of recombinant molecules mediated by repeated sequences of *C. carlesii* and *Ca. henryi*

Species	Repeat	Length (bp)	Reads span across regions	Reads support	Total reads support
*C. carlesii*	LR9	13,229	contig7-LR9-contig4	11	15 (57.69%)
			contig2-LR9-contig8	4	
			contig2-LR9-contig4	4	11 (42.31%)
			contig7-LR9-contig8	7	
	LR10	9,311	contig6-LR10-contig3	19	31 (56.36%)
			contig2-LR10-contig7	12	
			contig6-LR10-contig7	12	24 (43.64%)
			contig2-LR10-contig3	12	
	LR11	6,273	contig5-LR11-contig1	29	70 (56.45%)
			contig12-LR11-contig8	41	
			contig5-LR11-contig8	30	54 (43.55%)
			contig12-LR11-contig1	24	
*Ca. henryi*	LR7	2,876	contig1(ndh3)-LR7-contig5(ATG)	224	488(56.22%)
			contig5(CGG)-LR7-contig1(ndh9)	264	
			contig1(ndh3)-LR7-contig5(CCG)	172	380(43.78%)
			contig5(CAT)-LR7-contig1(ndh9)	208	
	LR8	650	contig4-LR8-contig3	80	176(22.31%)
			contig3-LR8-contig2	96	
			contig4-LR8-contig2	487	613(77.69%)
			contig3-LR8-contig3	126	
	LR9	481	contig2-LR9-contig6(TAA-TCC)-LR9-contig4	271(86.58%)	271(86.58%)
			contig2-LR9-contig6(GGA-TTA)-LR9-contig4	42(13.42%)	42(13.42%)

**Fig. 2:**
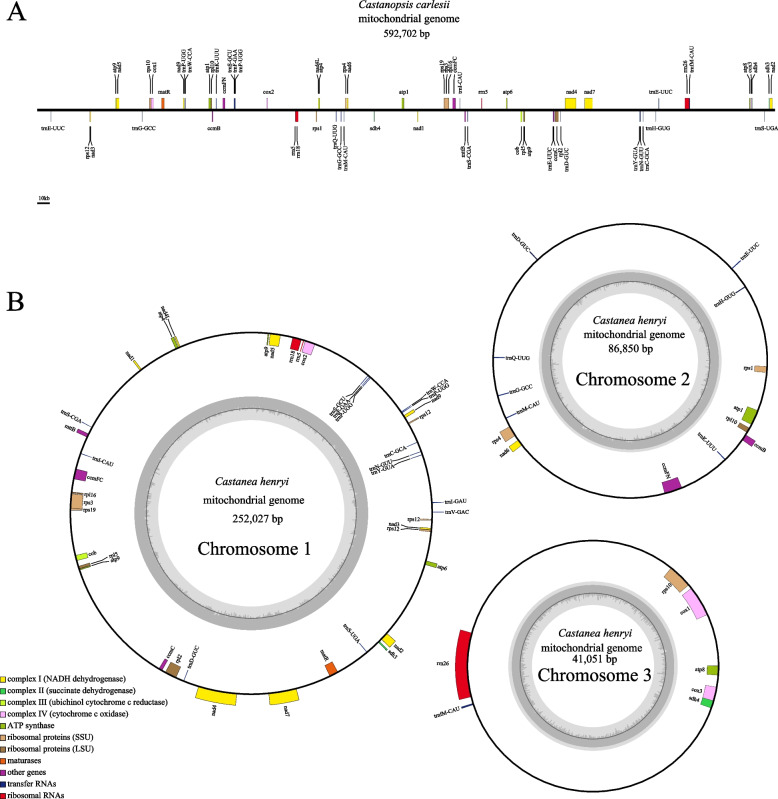
The map of the mitochondrial genome of *C. carlesii* (**A**) and *Ca. henryi* (**B**). Genes belonging to different functional groups are color-coded

For ease of description, based on the proportion of mitogenome recombination obtained, we processed the mitogenome of *C. carlesii* into a linear molecule with the sequence contig1-LR11-contig5-contig3-LR10-contig6-contig4-LR9-contig7-LR10-contig2-LR9-contig8-LR11-contig12 with a length of 592,702 bp (Fig. 2A). We processed the mtDNA1 of *Ca. henryi* into a circular molecule of chromosome 1 with the sequence contig1-LR7-contig5-LR7-contig1 with a length of 252,027 bp, processed mtDNA2 into a circular molecule of chromosome 2 with the sequence contig4-LR9-contig6-LR9-contig2-LR8-contig4 with a length of 86,851 bp and processed mtDNA3 into a circular molecule of chromosome 3 with the sequence contig3-LR8-contig3 with a length of 41,051 bp (Fig. 2B). It is important to note that this processing method is not exclusive as plant mtDNA structure can be influenced by dynamic transformations involving repetitive sequences. We chose this processing to facilitate subsequent analysis purposes.

### Repeat analysis

In the mitochondria of seven species of Fagaceae, we detected SSRs ranging in number from 143 to 203 (Table S2). The most abundant were tetranucleotide repeat sequences, accounting for 37.71% of the total SSRs. Mononucleotide repeat sequences accounted for 25.38% and dinucleotide repeat sequences accounted for 21.81% (Fig. S22A). Hexanucleotide repeat sequences were rare in these mitogenomes, with only one each found in *Q. variabilis* and *F. sylvatica* (Fig. S22B). Tandem repeat sequences ranging from 16 to 28 were detected in the mitochondria of seven species of Fagaceae, with lengths ranging from 785 bp to 1,423 bp (Fig. S22C, Table S3).

**Fig. 3:**
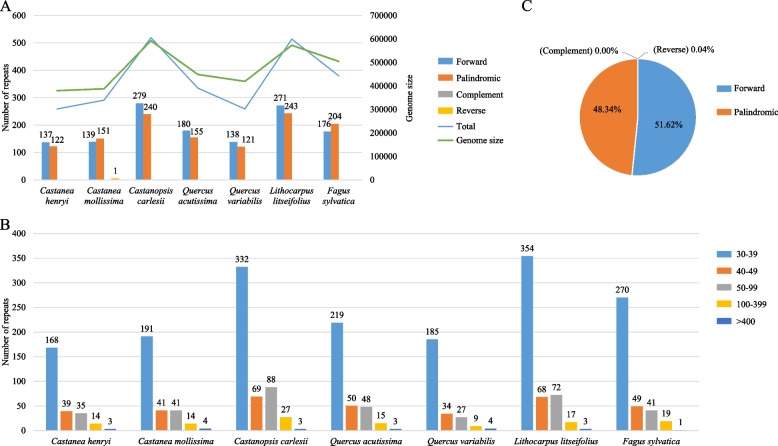
The type and presence of dispersed repeats among seven Fagaceae mitogenomes. **A** Types and number of dispersed repeats. **B** Number of dispersed repeats divided by length. **C** Percentage of dispersed repeats

The total number of four types of dispersed repeats (direct, palindromic, complementary and inverted repeats) varied from 259 to 519 across different species (Fig. 3A, Table S4). We classified all repeat sequences into five categories based on their length: 30–39 bp, 40–49 bp, 50–99 bp, 100–399 bp and ≥ 400 bp. Among these categories, those with a length of 30–39 bp were the most common, accounting for 67.23% of the total, while those longer than 400 bp were the least frequent at only 0.82% (Fig. 3B). The most abundant types of repeat sequences were the Forward and Palindromic ones, accounting for 51.62% and 48.34% of the total length of repeat sequences respectively. Only one Reverse repeat was found in *Ca. mollissima* (Fig. 3C).

### Codon usage and RNA editing prediction

We analyzed the unique PCGs in seven species of Fagaceae mitogenomes and calculated their codon usage frequency and RSCU values (Fig. S23, Table S5). The number of codons in these genes ranges from 10,109 (*F. sylvatica*) to 10,710 (*L. litseifolius*) (Table S5). We identified a total of 63 synonymous codons, excluding stop codons. Among these codons, 31 have RSCU values greater than 1, while another 31 have RSCU values less than 1, and only Tryptophan encoded by UGG has an RSCU value equal to 1 (Table S5). Leucine is the most encoded amino acid in all mitochondrial PCGs with a range of abundance between 10.50% and 10.64%, whereas cysteine is the least abundant at a range of abundance between 1.47% and 1.54% (Fig. S23).

The number of RNA editing sites in the mitogenome PCGs of various Fagaceae species ranges from 476 to 503. Among these, a total of 476 and 491 RNA editing events were identified in the mitochondrial PCGs of *C. carlesii* and *Ca. henryi*, respectively (Table S6). Notably, the *nad*4 gene exhibited the highest number of RNA editing events among all Fagaceae species mitochondrial PCGs, with 41 to 42 identified editing sites. On the other hand, no RNA editing events were found in the *rpl*2 gene (Fig. S24). Furthermore, we observed that amino acid changes at the second codon position accounted for most alterations at RNA editing sites across all seven Fagaceae species studied. The first codon position also contributed significantly to these changes, while minimal alterations occurred at both the first and second codon positions (Fig. S24). Most observed edits involved conversions from serine and proline to leucine; however, there were only a few instances where arginine was converted into a stop codon. Additionally, edits converting glutamine into a stop codon were exclusively identified in *F. sylvatica* (Fig. S24).

### Intracellular gene transfer from chloroplast to mitochondrial organelles

**Fig. 4:**
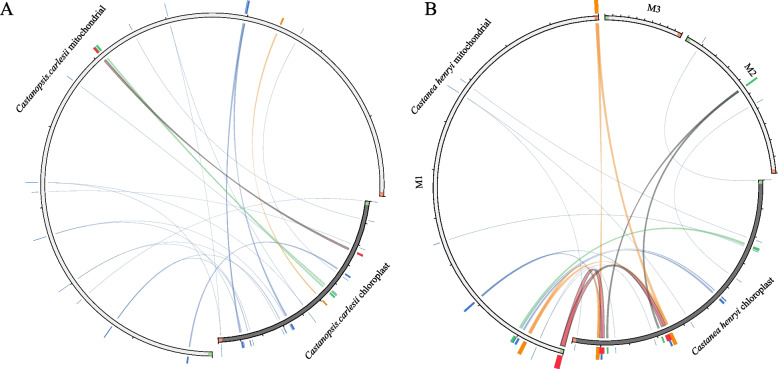
Homology analysis of mitogenome and chloroplast genome of *C. carlesii* (**A**) and *Ca. henryi* (**B**). The colored blocks outside the sequence describe the blast hit scoring, best quartile being red, then orange, green and blue, respectively

In the mitogenomes of *C. carlesii* and *Ca. henryi*, we identified 22 and 14 homologous segments shared with chloroplasts, respectively (excluding sequences aligned to chloroplast repeats) (Fig. 4, Table S7). We refer to these migrating fragments from chloroplasts to mitochondrial organelles as MTPTs. The length of the homologous fragments identified in the *C. carlesii* mitogenome ranged from 28 to 1147 bp. These MTPTs had a total length of 7086 bp, accounting for approximately 1.20% of the total mitogenome (Table S7). In the *Ca. henryi* mitogenome, the length of the MTPTs ranged from 74 to 2500 bp, with a total length of 10,608 bp representing approximately 2.79% of the total mitogenome (Table S7).

We annotated these homologous sequences and identified six complete genes in the *C. carlesii* mitogenome, including two PCGs (*pet*L and *psb*J) and seven tRNA genes (*trn*D-GUC, *trn*H-GUG, *trn*I-CAU, *trn*M-CAU, *trn*N-GUU, *trn*P-UGG and *trn*W-CCA). Similarly, five complete genes were found in *Ca. henryi* mitogenome, including two PCGs (*pet*L and *pet*G), one rRNA(*rrn*16), and eight tRNA genes (more *trn*V-GAC than *C. carlesii*). Additionally, *Q. variabilis* and *F. sylvatica* MTPTs were also identified (Table S7), as well as previous studies on *Ca. mollissima*, *L. litseifolius*, and *Q. acutissima*. We conducted a statistical analysis on the length, proportion, and complete genes of MTPTs in Fagaceae mitogenome (Table 4). It is evident that the total length of MTPTs in the seven species of Fagaceae ranges from 4589 bp to 19,904 bp, with their proportion relative to the total mitogenome length falling between 1.09% and 3.49% (Table 4). Among these fragments, we identified four complete tRNA genes, *trn*D-GUC, *trn*H-GUG, *trn*I-CAU, *trn*M-CAU, and *trn*N-GUU.

**Table 4:** The length, proportion, and contained full genes of MTPTs in *C. carlesii* and *Ca. henryi* mitogenomes

Species	Genome size	MTPTs Length (bp)	MTPTs proportion to total mitochondria (%)	Contained full genes		
				PCGs	rRNA genes	tRNA genes
*Ca. henryi*	379,929	10,608	2.79	*pet*G, *pet*L	*rrn*16	*trn*D-GUC, *trn*H-GUG, *trn*I-CAU, *trn*M-CAU, *trn*N-GUU, *trn*P-UGG, *trn*V-GAC, *trn*W-CCA
*Ca. mollissima*	388,038	5,766	1.49	/	/	*trn*D-GUC, *trn*H-GUG, *trn*I-CAU, *trn*M-CAU, *trn*N-GUU
*C. carlesii*	592,702	7,086	1.20	*pet*L, *psb*J	/	*trn*D-GUC, *trn*H-GUG, *trn*I-CAU, *trn*M-CAU, *trn*N-GUU, *trn*P-UGG, *trn*W-CCA
*Q. acutissima*	448,982	15,688	3.49	*pet*G, *pet*L	*rrn*16	*trn*A-UGC, *trn*D-GUC, *trn*H-GUG, *trn*I-CAU, *trn*I-GAU, *trn*M-CAU, *trn*N-GUU, *trn*P-UGG, *trn*V-GAC, *trn*W-CCA
*Q. variabilis*	419,744	4,589	1.09	*pet*G, *pet*L	/	*trn*D-GUC, *trn*H-GUG, *trn*M-CAU, *trn*N-GUU, *trn*N-GUU, *trn*P-UGG, *trn*W-CCA
*L. litseifolius*	573,177	19,904	2.08	/	*rn*18	*trn*D-GUC, *trn*H-GUG, *trn*I-GAU, *trn*M-CAU, *trn*N-GUU, *trn*P-UGG, *trn*V-GAC, *trn*W-CCA
*F. sylvatica*	504,715	5,733	1.14	*pet*G, *psb*E, *psb*F, *psb*J, *psb*L	/	*trn*D-GUC, *trn*H-GUG, *trn*I-CAU, *trn*M-CAU, *trn*N-GUU, *trn*P-UGG, *trn*W-CCA

### Features and comparison of mitogenomes

**Fig. 5:**
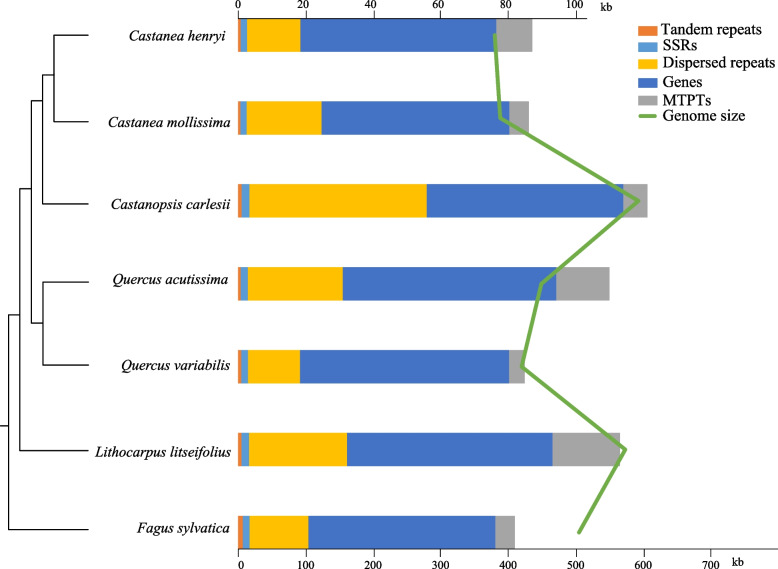
Genome size and content among seven Fagaceae mitogenomes. The genome size and the fraction of each genome covered by repeats, genes, MTPTs are shown. A schematic tree on the left shows the phylogenetic relationships among the species

During the lengthy evolutionary process of plants, there have been frequent changes in mitogenome size, GC content, and gene number. Among Fagales species (ranging from 379,929 bp to 719,418 bp), the differences in mitogenome size are relatively smaller compared to Cucurbitales species (ranging from 331,440 bp to 2,906,674 bp) (Table S1). The smallest mitogenome within Fagaceae is *Ca. henryi* with a size of 379,929 bp while the largest is *C. carlesii* with a size of 592,702 bp (Table S1). Our findings indicate that all species exhibit low proportions of tandem repeats and SSRs. There is no significant difference in gene length and the length of MTPTs does not correlate with mitogenome size. Dispersed repeats appear to be the main factor influencing the variation in mitogenome sizes within the Fagaceae family (Fig. 5). In terms of mitogenome GC content, Fagales species show higher values on average (45.59%) compared to Cucurbitales species (average 44.35%). Within Fagaceae species specifically, GC content ranges from 45.51% to 45.85%.

**Fig. 6:**
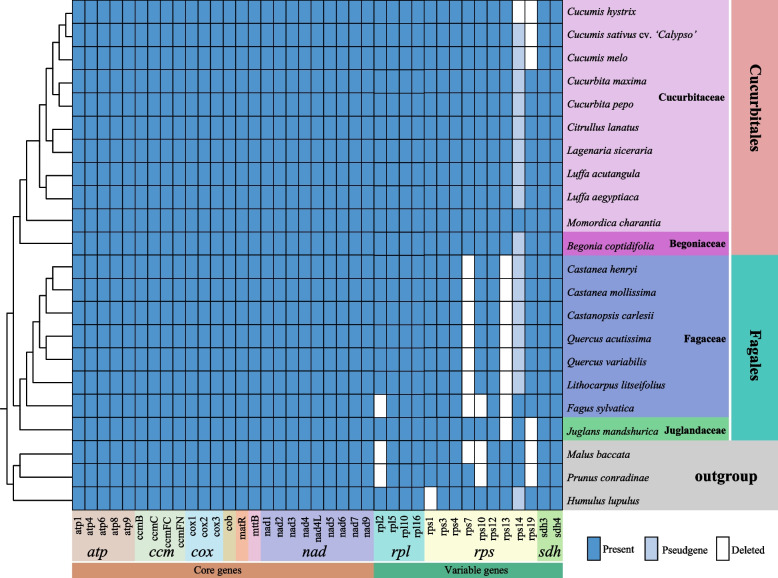
Distribution of PCGs in mitogenomes of *C. carlesii* and *Ca. henryi*. The blue box indicates that the gene exists, the light blue box indicates that the gene pseudgene, and a white box indicates the absence of the gene in the mitogenome

In terms of the number of genes in the mitogenome, both Cucurbitales and Fagales species have experienced varying degrees of gene loss. Core genes were not lost, while variable genes were lost to different extents (Fig. 6). In Cucurbitales, pseudogenization occurred at *rps*14 for variable genes, and loss mainly occurred at *rps*19. Only Momordica charantia did not experience any loss in its variable genes. On the other hand, all variable genes in Fagales species have been lost. The main lost genes are *rps*7 and *rps*13, with pseudogenization occurring in *rps*14 (Fig. 6). Interestingly, the pattern of gene loss on *F. sylvatica* differed from that of the other four genera within Fagaceae; there was a loss on *rpl*2 and *rps*10 but no pseudogenization on *rps*14 (Fig. 6). It is worth noting that only Fagales experienced a loss of *rps*13 among all groups studied (Fig. 6).

### Phylogenetic and synteny

**Fig. 7:**
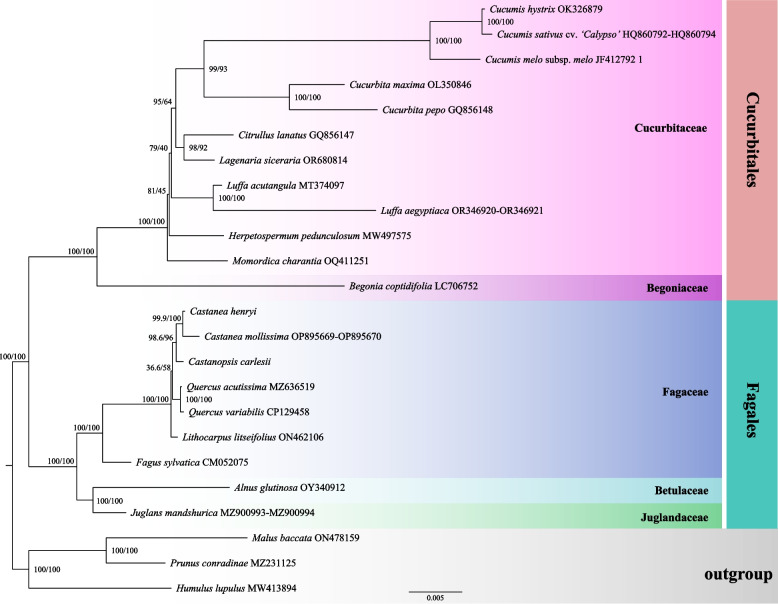
Maximum likelihood (ML) tree based on 39 PCGs. Numbers near the nodes are bootstrap percentages (SH-aLRT left, UFBoot right)

To determine the phylogenetic position of *C. carlesii* and *Ca. henryi* at the mitogenome level, we compared the mitogenomes of 19 species in Fagales and Cucurbitales, along with three species in Rosales as outgroups (Table S1). Based on 38 PCGs sequences, a phylogenetic tree was constructed (Fig. 7). The results revealed that Fagales as sister to Cucurbitales. Within Fagales, Fagaceae was found to be sister to Juglandaceae. In the Fagaceae branch, *F. sylvatica* emerged as sister to the other four genera. *L. litseifolius* showed a close relationship with *Castanea*, *Castanopsis* and *Quercus*, but had a short branch length and low support (SH-aLRT = 36.6, UFBoot = 58). *Ca. henryi* was identified as sister to *Ca. mollissima* followed by *C. carlesii*. Overall, except for *L. litseifolius* which has an unstable systematic position, the other genera have stable systematic positions.

**Fig. 8:**
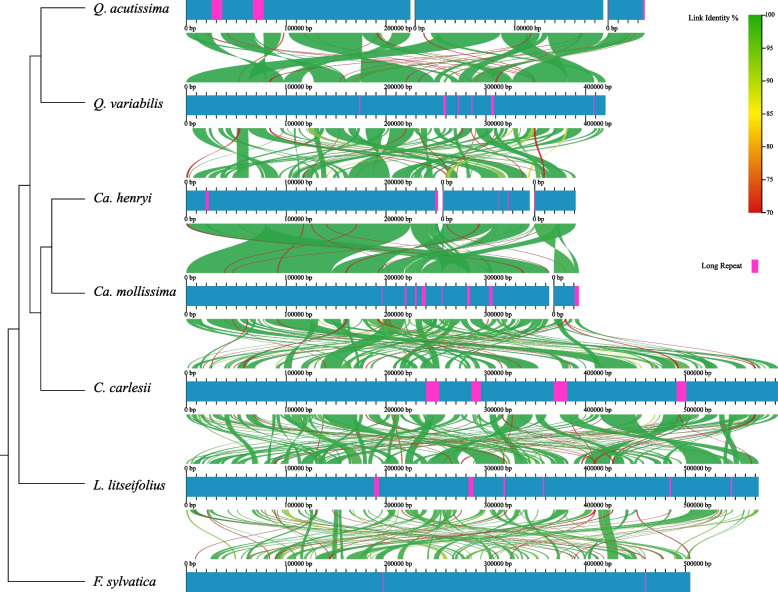
Collinear analysis of seven Fagaceae species. Arcs from red to green indicate that Link Identity ranges from 70 to 100%. The pink area is Long Repeat. A schematic tree on the left shows the phylogenetic relationships among the species

To further investigate the collinearity of mitogenomes in the Fagaceae, we utilized AliTV to visualize the mitogenome structure of seven Fagaceae species. The results revealed a generally conserved gene homology (Fig. 8). It was observed that the mitogenomes of Fagaceae exhibit conserved gene homology and contain numerous homologous collinear segments that span most regions of the mitogenome. However, these homologous collinear segments vary in length, with closer related species within the same genus (*Quercus* and *Castanea*) having longer homologous collinear segments (Fig. 8).

## Discussion

Mitochondria are the cellular energy source for life processes, and their genomes are complex, exhibiting extensive sequence variations [19]. Plant mitogenomes are usually assembled and shown as circular maps, but plant mtDNA most likely does not exist as a typical circular structure, but rather as a complex dynamic collection of predominantly linear DNA combined with smaller circular and branched DNA molecules [18–20]. The mitogenome of Fagaceae plants displays various morphological characteristics. For example, the mitogenome of *Ca. mollissima* has a polycyclic structure consisting of two circular molecules [22], while the mitogenome of *Q. acutissima* is composed of three branched structures, consisting of one linear molecule and two circular molecules [21]. In contrast, the mitogenomes of *L. litseifolius* and *F. sylvatica* are assembled into classic circular genomes [23, 49]. In this study, the mitogenome of *C. carlesii* showed a branched structure, while the mitogenome of *Ca. henryi* showed a polycyclic structure, consisting of three circular chromosome molecules.

Large repetitive sequences are commonly found in the mitogenomes of angiosperms, typically occurring in 2 to 3 copies and often mediate homologous recombination to form the characteristic multi-part structure of angiosperm mitogenomes [24, 25]. Recent studies of the mitochondrial genomes of the Fagaceae family using advancements in long-read sequencing technology have revealed the crucial role of repetitive sequences in mediating homologous recombination and allopolyploidy [21, 22]. The size of repetitive sequences is closely associated with recombination frequency, with longer repetitive sequences generally exhibiting higher rates of recombination than shorter ones. Additionally, long repetitive sequences contribute more evenly to allopolyploidy proportions [50]. During our analysis of the *C. carlesii* and *Ca. henryi* mitogenomes, we identified three pairs of repetitive sequences with recombination activity in each species. Among these, four longer repetitive sequences (over 2,800 bp) exhibited high recombination frequencies and contributed to allopolyploidy with a ratio of approximately 4:6. In contrast, two shorter repetitive sequences (481 bp and 650 bp) identified in *Ca. henryi* had lower recombination frequencies and resulted in significantly different allopolyploidy proportions, with a ratio of approximately 1:9 for one sequence and 1:4 for the other.

In addition to large repetitive sequences, mitogenomes also include extensive tandem repetitive sequences and short repetitive sequences. These sequences not only facilitate genome rearrangement but also contribute significantly to mtDNA size expansion [51, 52]. The size of angiosperm mitogenomes varies greatly, ranging from 66 Kb to 11.3 Mb, with known genes between 19 and 64 [16, 17]. This wide range in genome size can be attributed to variations in the proportions of repetitive DNA, gene-interspersed regions, and exogenous sequences [51]. For example, a study on a nearly 1 Mb *Cucurbita pepo* mitogenome found that its size was primarily due to the proliferation of abundant exogenous chloroplast sequences and small repetitive sequences [53], while another study on a cucumber (*Cucumis sativus*) mitogenome measuring approximately 1685 kb revealed that its larger size resulted mainly from dispersed repetitive sequence proliferation, intron expansion, and acquisition of external sequences [54]. The *C. carlesii* mitogenome amounts to 592,702 bp, comparable to that of the *L. litseifolius* mitogenome (573,177 bp) [23]. This makes it the largest mitogenome discovered in the Fagaceae family thus far. The total length of the *Ca. henryi* mitogenome is 379,929 bp, close to the size of the 388,038 bp mitogenome of *Ca. mollissima* in the same genus [22] and the 448,982 bp mitogenome of *Q. acutissima* in the *Quercus* genus [21] and the 419,744 bp mitogenome of *Q. variabilis* (CP129458). This makes it the smallest mitogenome discovered in the Fagaceae family thus far. To investigate factors contributing to variation in mitogenome size within the Fagaceae family, we conducted statistical analysis on tandem repeats, SSRs, dispersed repeats, genes and MTPTs present in each species' mitogenomes (Fig. 6). Our findings indicate that dispersed repeats are primarily responsible for variations in mitogenome size within this family while MTPTs do not play a significant role.

In angiosperms, the loss and transfer of mitochondrial genes are frequent and ongoing phenomena. Currently, almost all mitogenomes of angiosperms have a complete set of 24 core genes, while 14 ribosomal protein genes and two *sdh* genes have been lost between 6 to 42 times [55]. In the mitogenomes of Cucurbitales and Fagales, all 24 core genes were found to be present, but there were variations in gene losses. Among the mitogenomes of seven Fagaceae species, except for *F. sylvatica*, the variable gene losses include *rps*7 and *rps*13, with pseudogene losses including *rps*14. Recent studies have shown that *F. sylvatica* exhibits a different pattern of variable gene loss compared to the other four genera in Fagaceae [56]. This difference may be attributed to the longer divergence time between *Fagus* and the other four genera resulting in distinct patterns of gene loss during evolution. Although *rps*7 is the most frequently lost ribosomal protein gene, it appears to be rarely functionally transferred to the nucleus [55, 57]. Many plant mitogenomes co-transcribe *rps*14 with *rpl*5 and *cob* [58, 59]. In some lineages such as *Arabidopsis thaliana*, a copy of *rps*14 has been relocated to the nucleus while its mitochondrial counterpart becomes a pseudogene [60–62]. In Fagaceae, the mitogenome contains a pseudogene for *rps*14 located between *rpl*5 and *cob* genes. Thus, Fagaceae likely experienced a relocation event where *rps*14 was transferred to the nucleus, while the mitochondrial copy becoming a pseudogene. Previous studies have reported losses of *rps*13 in 24 out of 84 rosids and one loss in 77 asterids. It is possible that many losses of *rps*13 in Rosaceae plants can be traced back to a single event: replication of chloroplast *rps*13 gene by nuclear genes and replacement of mitochondrial *rps*13 gene products [55]. In our study, the loss of *rps*13 was exclusively observed in the Fagales, potentially representing a single gene substitution event in the common ancestor of rosids. Most losses of mitochondrial genes are likely due to their transfer to the nucleus [63], thus further investigation is needed to determine if missing genes in Fagaceae mitogenomes have been transferred to the nucleus.

There are 64 codons present in eukaryotic genomes, and the usage rate of these codons varies significantly among different species and organisms. This variation is believed to be the result of a balanced evolutionary selection process that has occurred within cells over an extended period. We have identified a total of 63 codons, excluding the stop codon. Among these codons, 31 have an RSCU value greater than 1, while 31 have an RSCU value less than 1. The UGG codon is the only one that encodes Trp = 1. Leu is the most commonly encoded amino acid across all mitochondria, accounting for approximately 10.48% to 10.64% of its composition. On the other hand, Cys has the lowest content at around 1.47% to 1.54%. RNA editing is a post-transcriptional event that occurs in plant organelles [64]. It serves as an indirect repair mechanism capable of correcting DNA mutations at the RNA level [65]. Most RNA editing events in plant organelles originate from C-to-U conversion at specific sites [66]. Previous studies have identified 484 RNA editing sites in 35 PCGs of *Ca. mollissima* [22], 466 sites in 32 PCGs of *Q. acutissima* [21], and a total of 461 sites in the PCGs of *L. litseifolius* [23]. In the mitogenomes of *C. carlesii* and *Ca. henryi*, we have identified 476 and 491 editing sites, respectively, in their respective set of 36 PCGs. Within the Fagaceae, serine and proline to leucine conversions are most common among the editing events observed. The number of RNA editing sites varies greatly among different genes, with *nad*4 exhibiting the highest frequency while *rpl*2 does not show any RNA editing events. Previous studies have indicated that half of all RNA editing occurs at the second codon position [23, 67]. Consistent with these findings, we have observed that approximately between 59.27% to 60.29% of our detected editing sites occur at this second position.

Throughout the evolution of higher plants, intergenomic gene transfer between organelle genomes has been significant [68]. With the sequencing of numerous plant mitochondrial and plastid genomes, it has been discovered that MTPTs are a common feature in angiosperm mitogenomes. In most plants, plastid DNA in the mitogenome ranges from about 1% to 10% [69]. The identified MTPTs in the *C. carlesii* mitogenome account for about 1.20%, while those in the *Ca. henryi* mitogenome account for approximately 2.79%. By determining the proportion of MTPTs in *Q. variabilis* and *F. sylvatica* mitogenomes, as well as statistically examining the published mitogenomes MTPTs results of *Ca. mollissima* [22], *L. litseifolius* [23], and *Q. acutissima* [21], we obtained a range of MTPTs accounting for approximately 1.09–3.49% of total mitogenome length within Fagaceae. In the medium-sized genome of *Cucurbita pepo*, there are 113 kb (11.5%) of MTPTs, which is the highest proportion recorded among all sequenced plants to date [53]. In contrast, the large genome of melon (*Cucumis melo*) with a size of 2.74 Mb only contains 39 kb of MTPTs, representing a very low proportion (1.4%) [70]. However, in this study, the mitogenome size of *C. carlesii* is 592,702 bp with an MTPTs proportion of 1.20%, while *Ca. henryi* has a mitogenome size of 379,929 bp with a higher MTPTs proportion at 2.79%. This indicates that the difference in mitogenome size does not correlate with the proportion of MIPTs in them. The transfer of tRNA gene sequences from chloroplast to mitogenomes is common in plants [71]. Among the seven mitogenomes studied within the Fagaceae family, we identified four complete chloroplast genes: *trn*D-GUC, *trn*H-GUG, *trn*I-CAU, *trn*M-CAU, and *trn*N-GUU.

In this study, we conducted a further analysis of the phylogenetic relationship of *C. carlesii* and *Ca. henryi* based on the mitogenome. The findings revealed that *Ca. henryi* is sister to the same genus of *Ca. mollissima*, while *C. carlesii* of *Castanopsis* was demonstrated to be sister to *Castanea*. *F. sylvatica* was positioned at the base of the Fagaceae and was shown to be sister to the other four genera. *L. litseifolius* was closely related to *Castanea*, *Castanopsis*, and *Quercus*, but its position received relatively low support. These results resemble the previous phylogenetic tree constructed using plastid genomes [72], which might be attributed to the fact that mitochondrial genomes and plastid genomes share the same genetic background. Through in-depth exploration of the alignment relationships of seven mitogenome sequences, including *C. carlesii*, *Ca. henryi*, *Ca. mollissima*, *F. sylvatica*, *L. litseifolius*, *Q. acutissima*, and *Q. variabilis*, a considerable number of homologous collinear fragments of varying lengths were identified. Species within the same genus, such as *Quercus* and *Castanea*, possess longer homologous collinear segments. These mitochondrial genomes of more closely related species exhibit a lower degree of mitochondrial genome segregation during the evolutionary process. These discoveries indicate that the mitotic genomes of species within the Fagaceae family have undergone extensive rearrangements.

## Conclusion

This study used Illumina and PacBio sequencing technologies for the first time to successfully assemble the complete mitogenome of *Castanopsis carlesii* and *Castanea henryi*, revealing the unique conformation in their mitogenome structures. The mitogenome of *C. carlesii* has a multi-branched structure with a total length of 592,702 bp, while the mitogenome of *Ca. henryi* has a three circular chromosome structure with a total length of 379,929 bp. This study shows that the main factor affecting the size difference of mitogenome in Fagaceae is dispersed repeats. Additionally, we found that the chloroplast sequence transfer ranges from 1.09% and 3.49% in Fagaceae mitogenome, while MTPTs insertion does not correlate with mitogenome size. This study enhances the understanding of Fagaceae mitogenome and provides a reference for future evolutionary research on the mitochondrial genome of this family.

## Supplementary Information

Supplementary Material 1.

Supplementary Material 2.

Supplementary Material 3.

Supplementary Material 4.

Supplementary Material 5.

Supplementary Material 6.

Supplementary Material 7.

Supplementary Material 8.

## Data Availability

The accession number of *Castanopsis carlesii* mitogenome in GeneBank is PP853255, and the accession number of *Castanea henryi* mitogenome in GeneBank is PP856681-PP856683.

## Abbreviations

tRNA: Transfer RNA
rRNA: Ribosomal RNA
SSR: Simple sequence repeat
CTAB: Cetyltrimethylammonium bromide
RSCU: Relative synonymous codon usage ratios
mtDNA: Mitochondrial DNA
MTPTs: Mitochondrial plastid DNA segments
SMRT: Single-molecule real-time
PCG: Protein-coding gene
