# Supplementary material for: The complete mitochondrial genome of Castanopsis carlesii and Castanea henryi reveals the rearrangement and size differences of mitochondrial DNA molecules
Source: BMC Plant Biol. 2024 Oct 21;24:988. doi: 10.1186/s12870-024-05618-z (PMC11492686; doi:10.1186/s12870-024-05618-z)
Supplement: Supplementary file 1 — Supplementary Material 1. [file 12870_2024_5618_MOESM1_ESM.docx]

**Supplementary Material 1**

[**Supplementary Figure S1.** The mapping results of the regions including the contig2-LR9-contig4 sequences in *C. carlesii* mitogenome. 3](#_Toc173943309)

[**Supplementary Figure S2.** The mapping results of the regions including the contig7-LR9-contig8 sequences in *C. carlesii* mitogenome. 3](#_Toc173943310)

[**Supplementary Figure S3.** The mapping results of the regions including the contig2-LR9-contig8 sequences in *C. carlesii* mitogenome. 4](#_Toc173943311)

[**Supplementary Figure S4.** The mapping results of the regions including the contig7-LR9-contig4 sequences in *C. carlesii* mitogenome. 4](#_Toc173943312)

[**Supplementary Figure S5.** The mapping results of the regions including the contig2-LR10-contig3 sequences in *C. carlesii* mitogenome. 5](#_Toc173943313)

[**Supplementary Figure S6.** The mapping results of the regions including the contig6-LR10-contig7 sequences in *C. carlesii* mitogenome. 5](#_Toc173943314)

[**Supplementary Figure S7.** The mapping results of the regions including the contig2-LR10-contig7 sequences in *C. carlesii* mitogenome. 6](#_Toc173943315)

[**Supplementary Figure S8.** The mapping results of the regions including the contig6-LR10-contig3 sequences in *C. carlesii* mitogenome. 6](#_Toc173943316)

[**Supplementary Figure S9.** The mapping results of the regions including the contig5-LR11-contig1 sequences in *C. carlesii* mitogenome. 7](#_Toc173943317)

[**Supplementary Figure S10.** The mapping results of the regions including the contig12-LR11-contig8 sequences in *C. carlesii* mitogenome. 7](#_Toc173943318)

[**Supplementary Figure S11.** The mapping results of the regions including the contig5-LR11-contig8 sequences in *C. carlesii* mitogenome. 8](#_Toc173943319)

[**Supplementary Figure S12.** The mapping results of the regions including the contig12-LR11-contig1 sequences in *C. carlesii* mitogenome. 8](#_Toc173943320)

[**Supplementary Figure S13.** The mapping results of the regions including the contig1-LR7-contig5 sequences in *Ca. henryi* mitogenome. 9](#_Toc173943321)

[**Supplementary Figure S14.** The mapping results of the regions including the contig5-LR7-contig1 sequences in *Ca. henryi* mitogenome. 9](#_Toc173943322)

[**Supplementary Figure S15.** The mapping results of the regions including the contig1-LR7-contig5 sequences in *Ca. henryi* mitogenome. 10](#_Toc173943323)

[**Supplementary Figure S16.** The mapping results of the regions including the contig5-LR7-contig1 sequences in *Ca. henryi* mitogenome. 10](#_Toc173943324)

[**Supplementary Figure S17.** The mapping results of the regions including the contig4-LR8-contig3 and contig3-LR8-contig2 sequences in *Ca. henryi* mitogenome. 11](#_Toc173943325)

[**Supplementary Figure S18.** The mapping results of the regions including the contig4-LR8-contig2 sequences in *Ca. henryi* mitogenome. 11](#_Toc173943326)

[**Supplementary Figure S19.** The mapping results of the regions including the contig3-LR8-contig3 sequences in *Ca. henryi* mitogenome. 12](#_Toc173943327)

[**Supplementary Figure S20.** The mapping results of the regions including the contig2-LR9-contig6 and contig6-LR9-contig4 sequences in *Ca. henryi* mitogenome. 12](#_Toc173943328)

[**Supplementary Figure S21.** The mapping results of the regions including the contig2-LR9-contig6 and contig6-LR9-contig4 sequences in *Ca. henryi* mitogenome. 13](#_Toc173943329)

[**Supplementary Figure S22.** Analyses of SSR and tandem repeats among seven Fagaceae mitogenomes 14](#_Toc173943330)

[**Supplementary Figure S23.** Amino acid frequencies among seven Fagaceae mitogenomes PCGs. 15](#_Toc173943331)

[**Supplementary Figure S24.** Comparison of RNA editing sites among seven Fagaceae mitogenomes PCGs 15](#_Toc173943332)


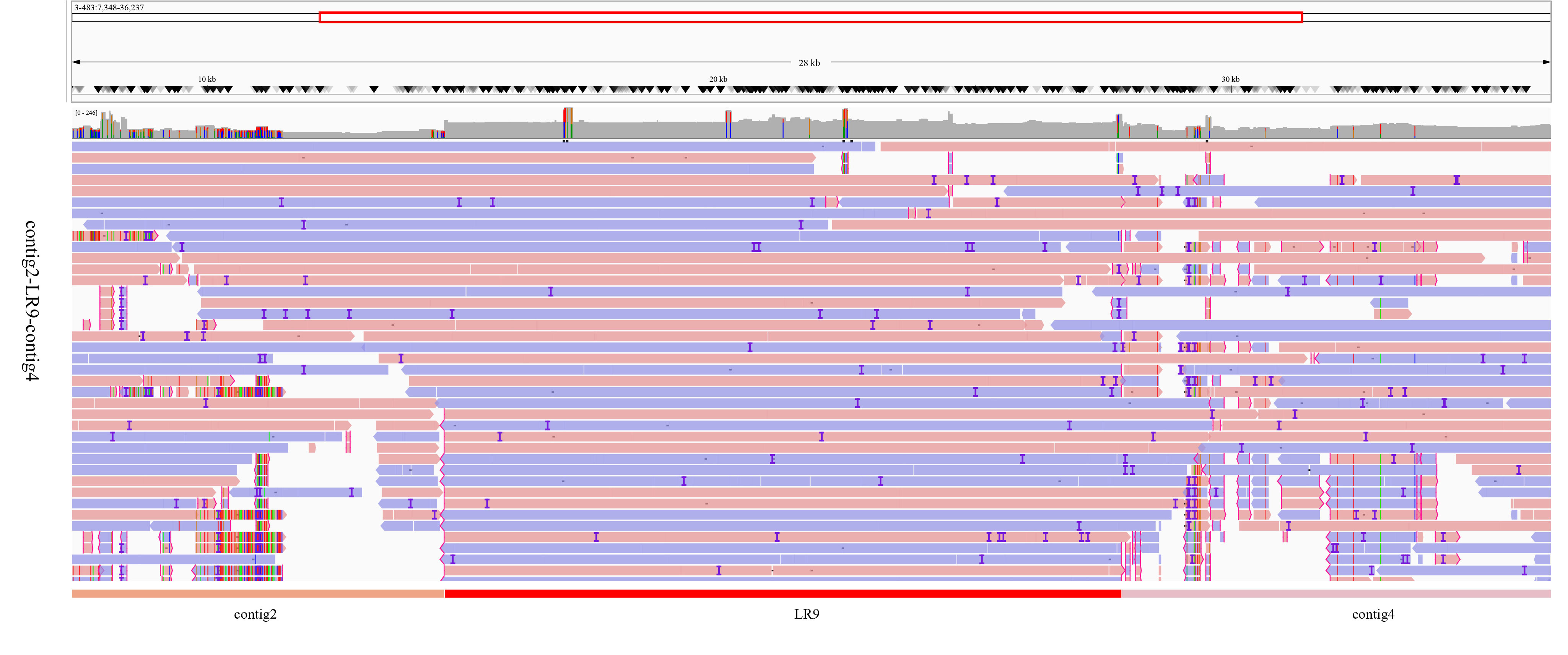


**Supplementary Figure S1.** The mapping results of the regions including the contig2-LR9-contig4 sequences in *C. carlesii* mitogenome.


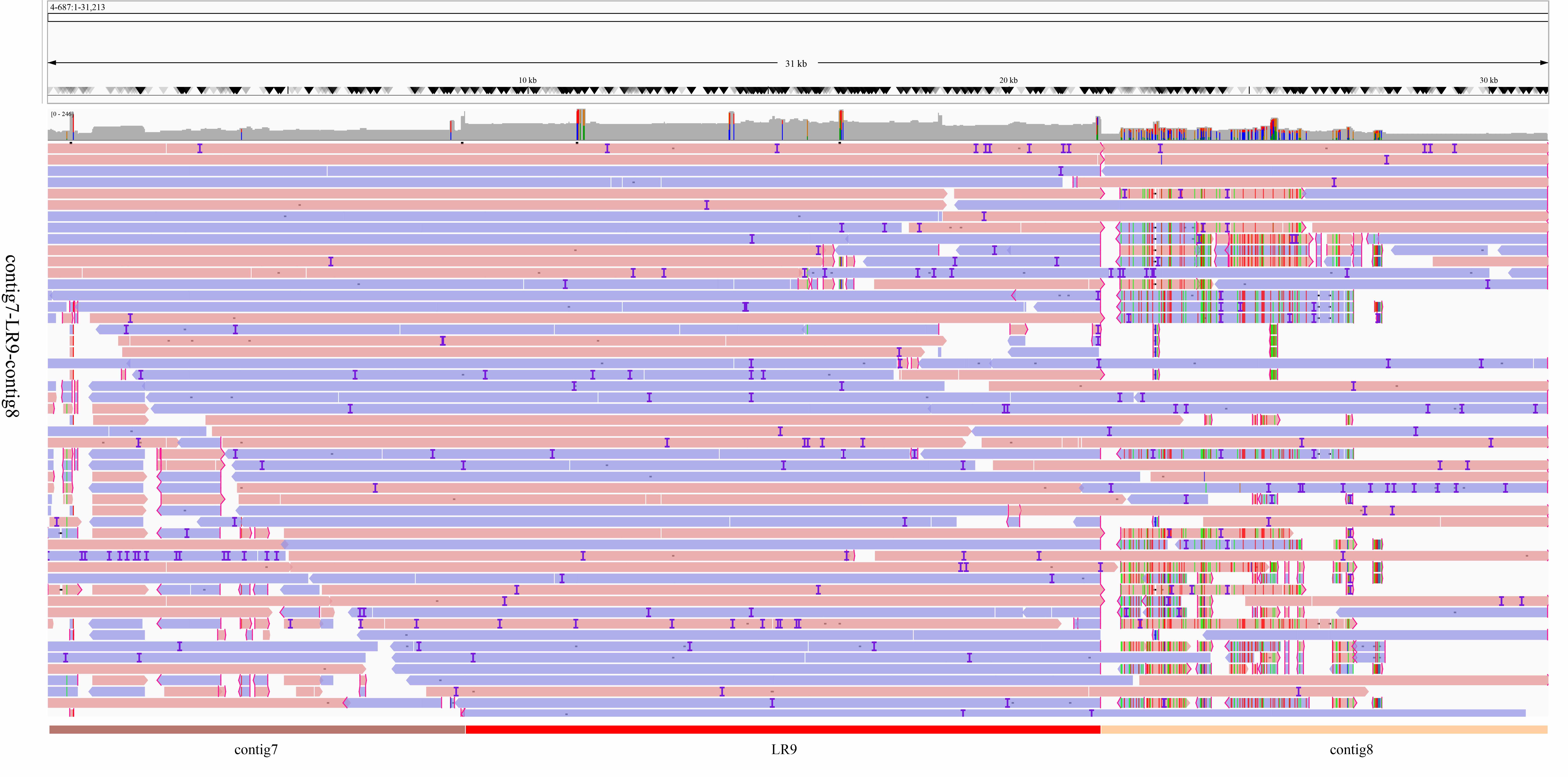


**Supplementary Figure S2.** The mapping results of the regions including the contig7-LR9-contig8 sequences in *C. carlesii* mitogenome.


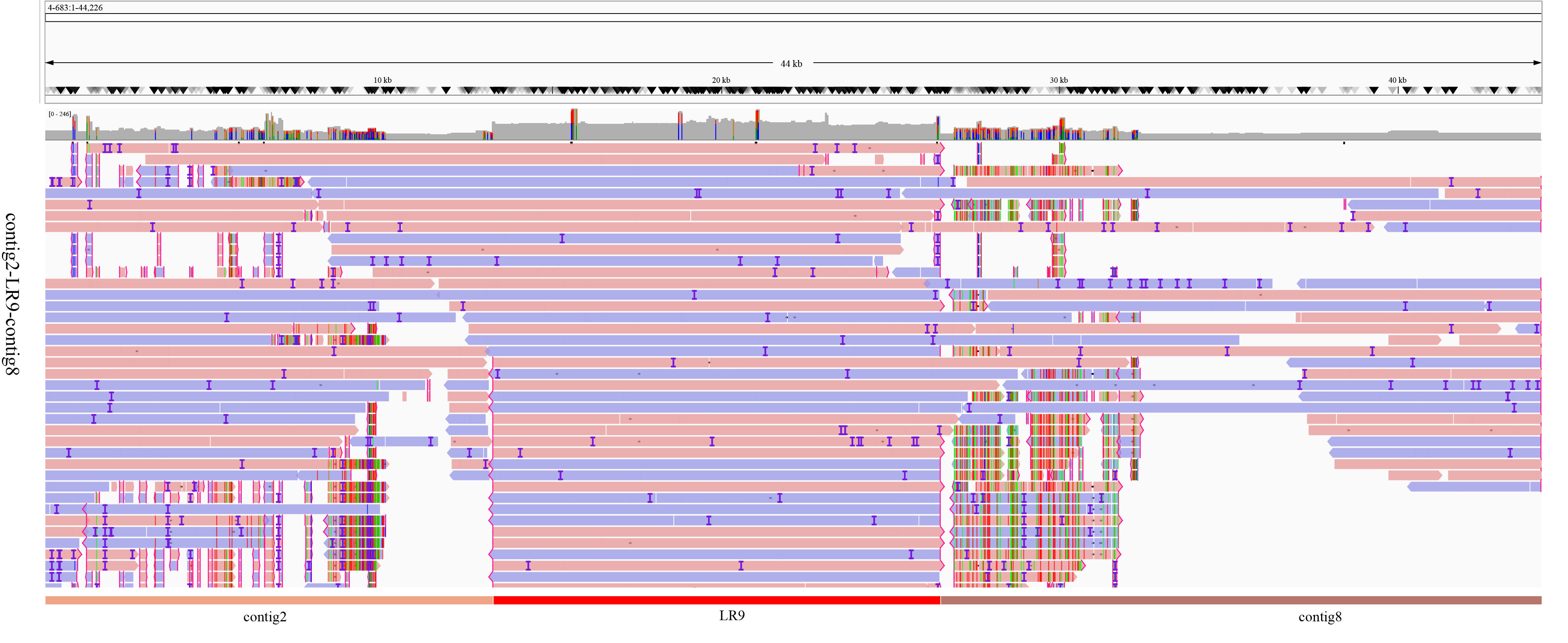


**Supplementary Figure S3.** The mapping results of the regions including the contig2-LR9-contig8 sequences in *C. carlesii* mitogenome.


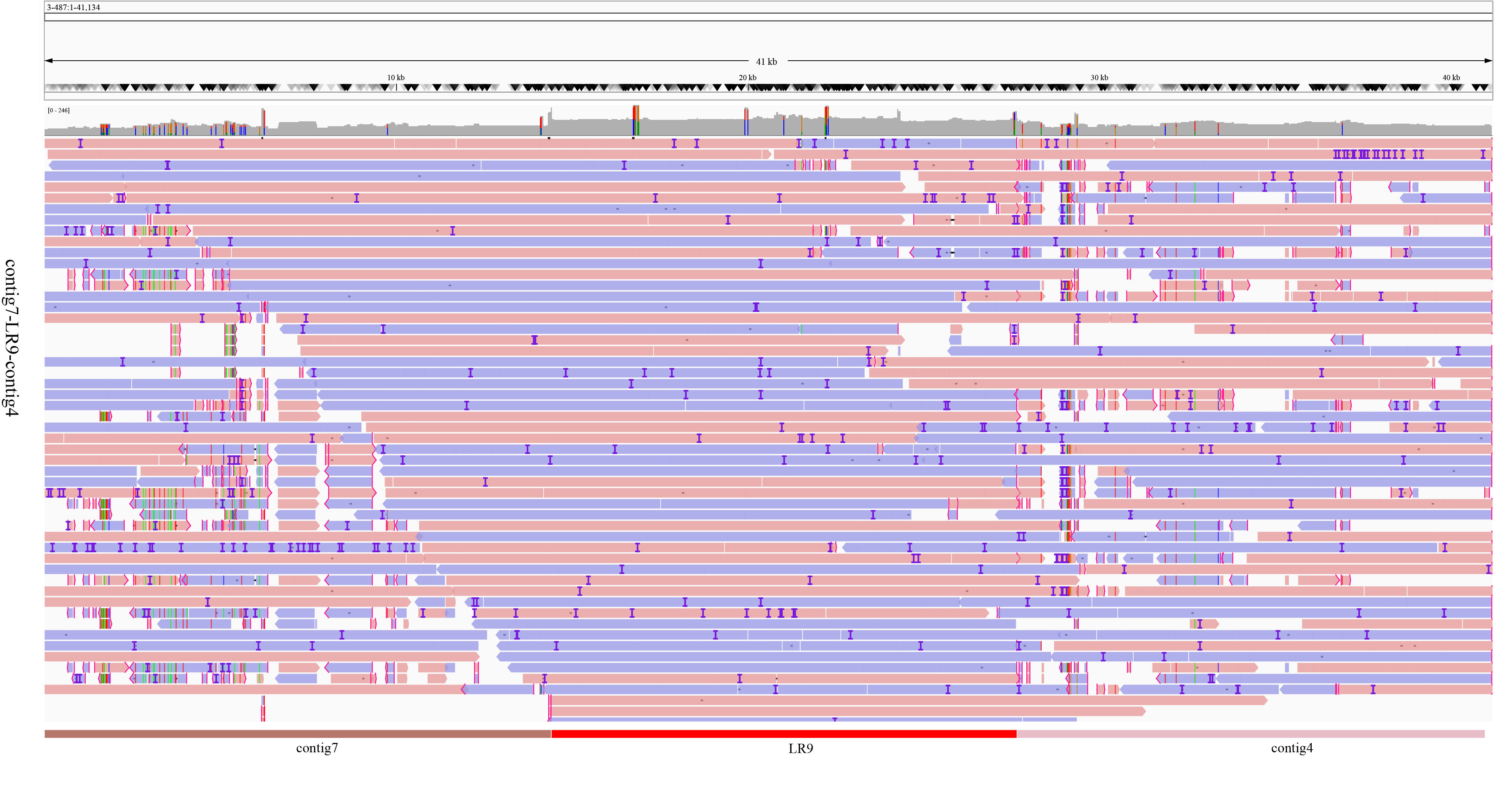


**Supplementary Figure S4.** The mapping results of the regions including the contig7-LR9-contig4 sequences in *C. carlesii* mitogenome.


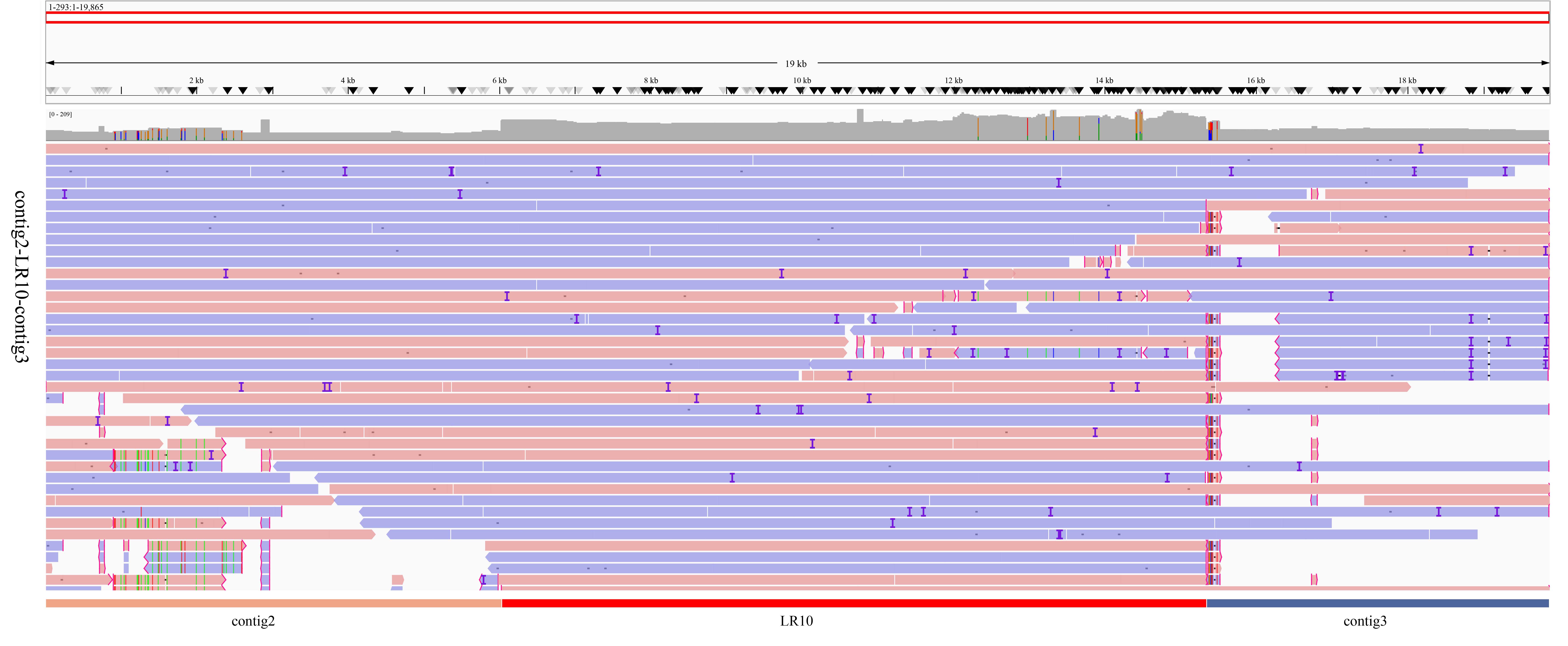


**Supplementary Figure S5.** The mapping results of the regions including the contig2-LR10-contig3 sequences in *C. carlesii* mitogenome.


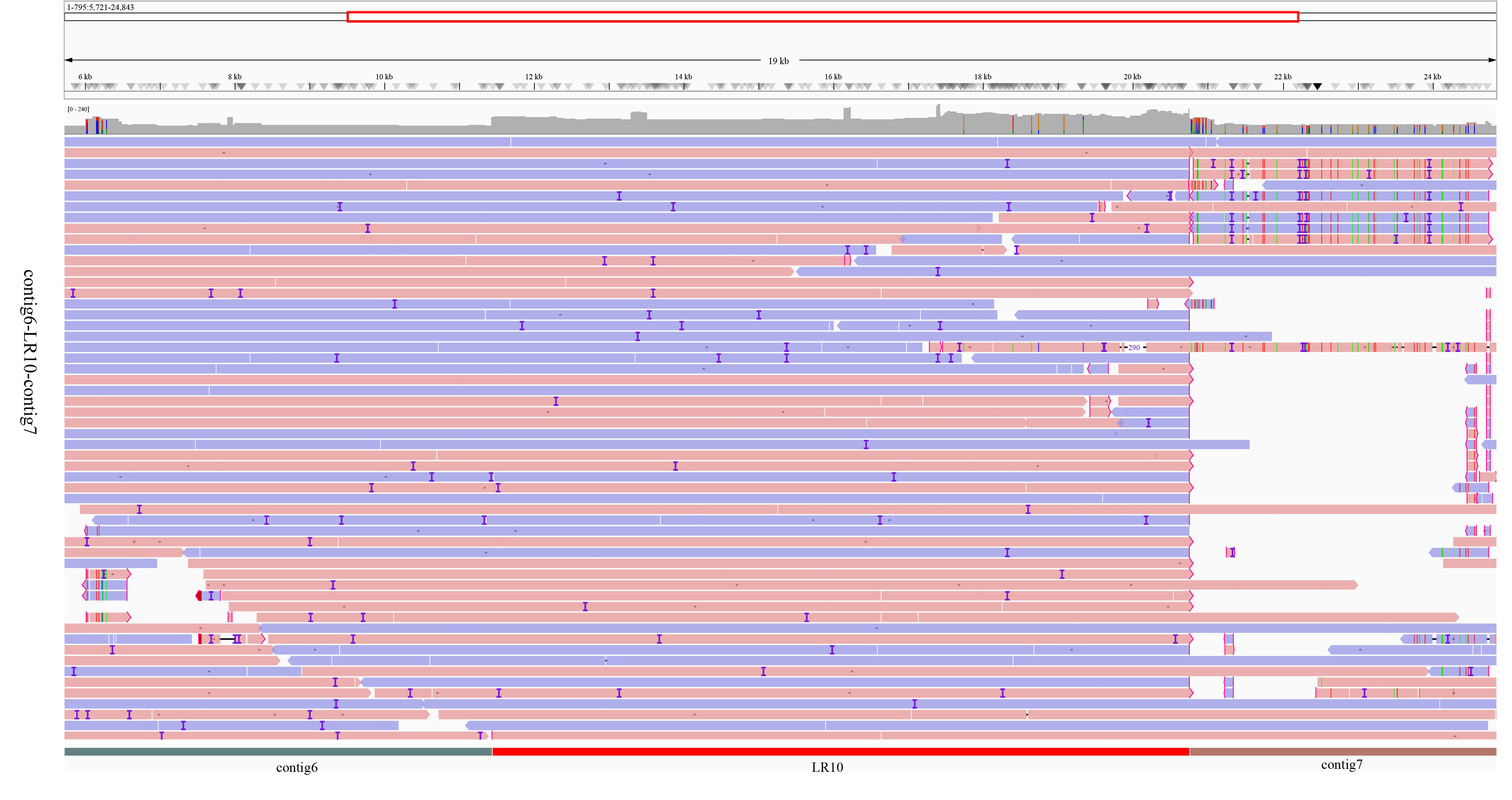


**Supplementary Figure S6.** The mapping results of the regions including the contig6-LR10-contig7 sequences in *C. carlesii* mitogenome.


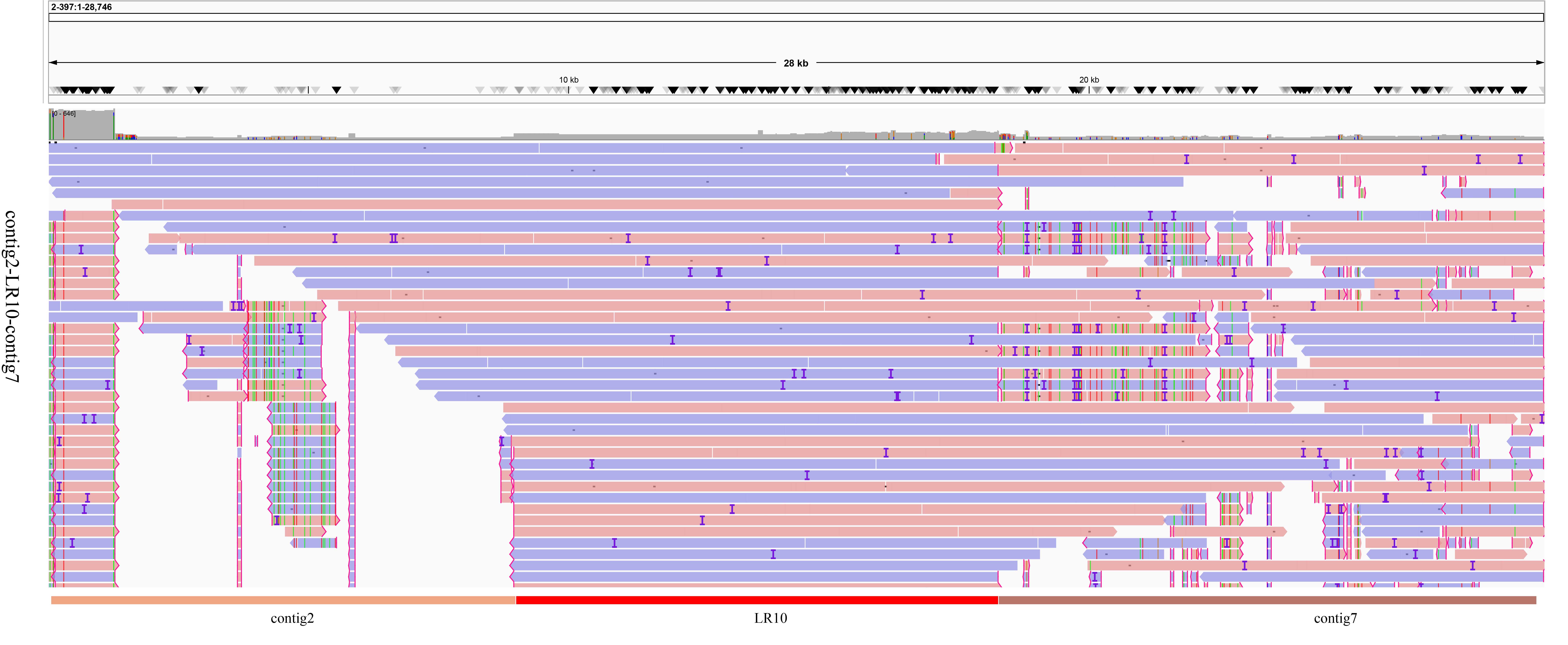


**Supplementary Figure S7.** The mapping results of the regions including the contig2-LR10-contig7 sequences in *C. carlesii* mitogenome.


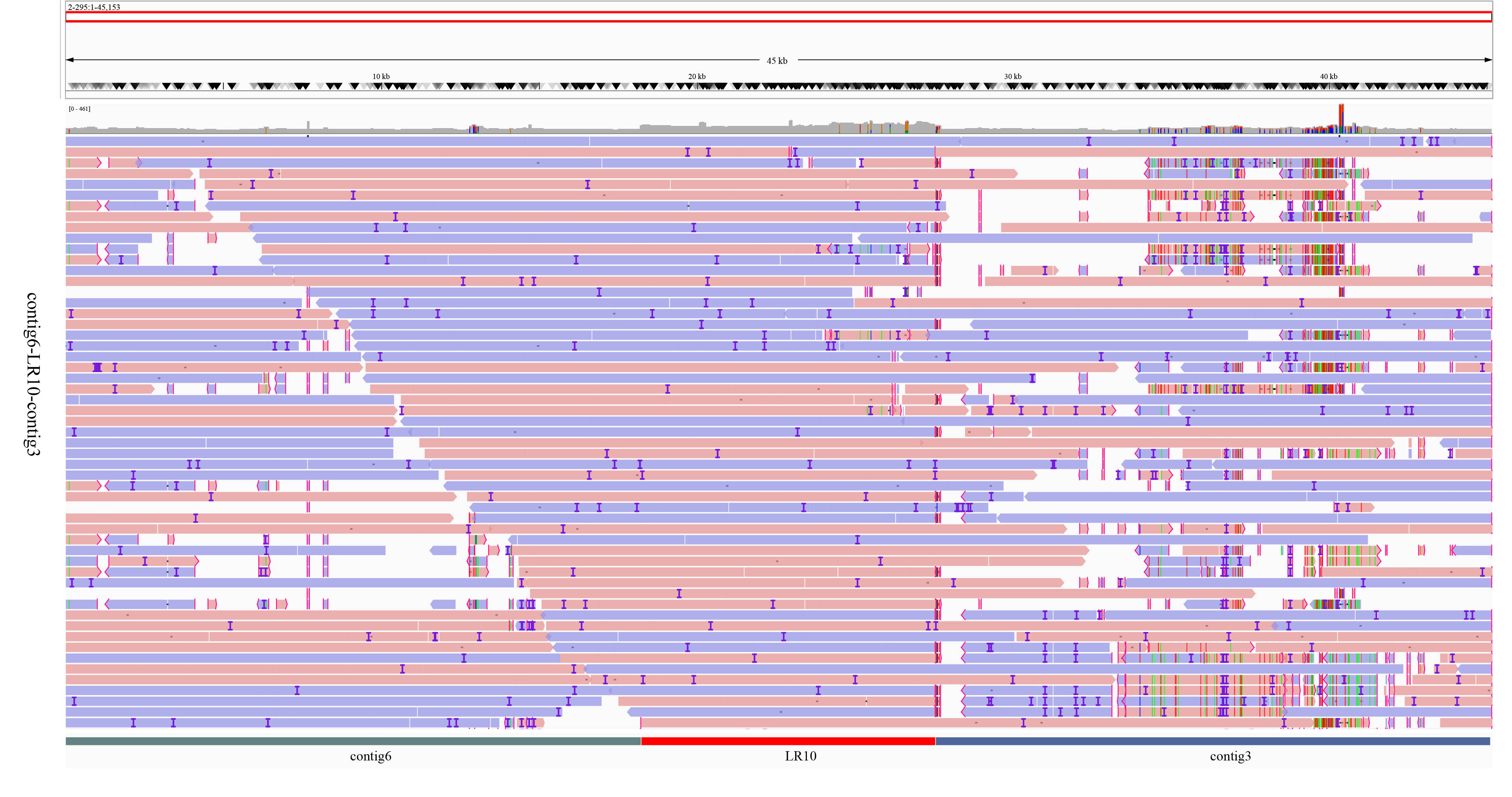


**Supplementary Figure S8.** The mapping results of the regions including the contig6-LR10-contig3 sequences in *C. carlesii* mitogenome.


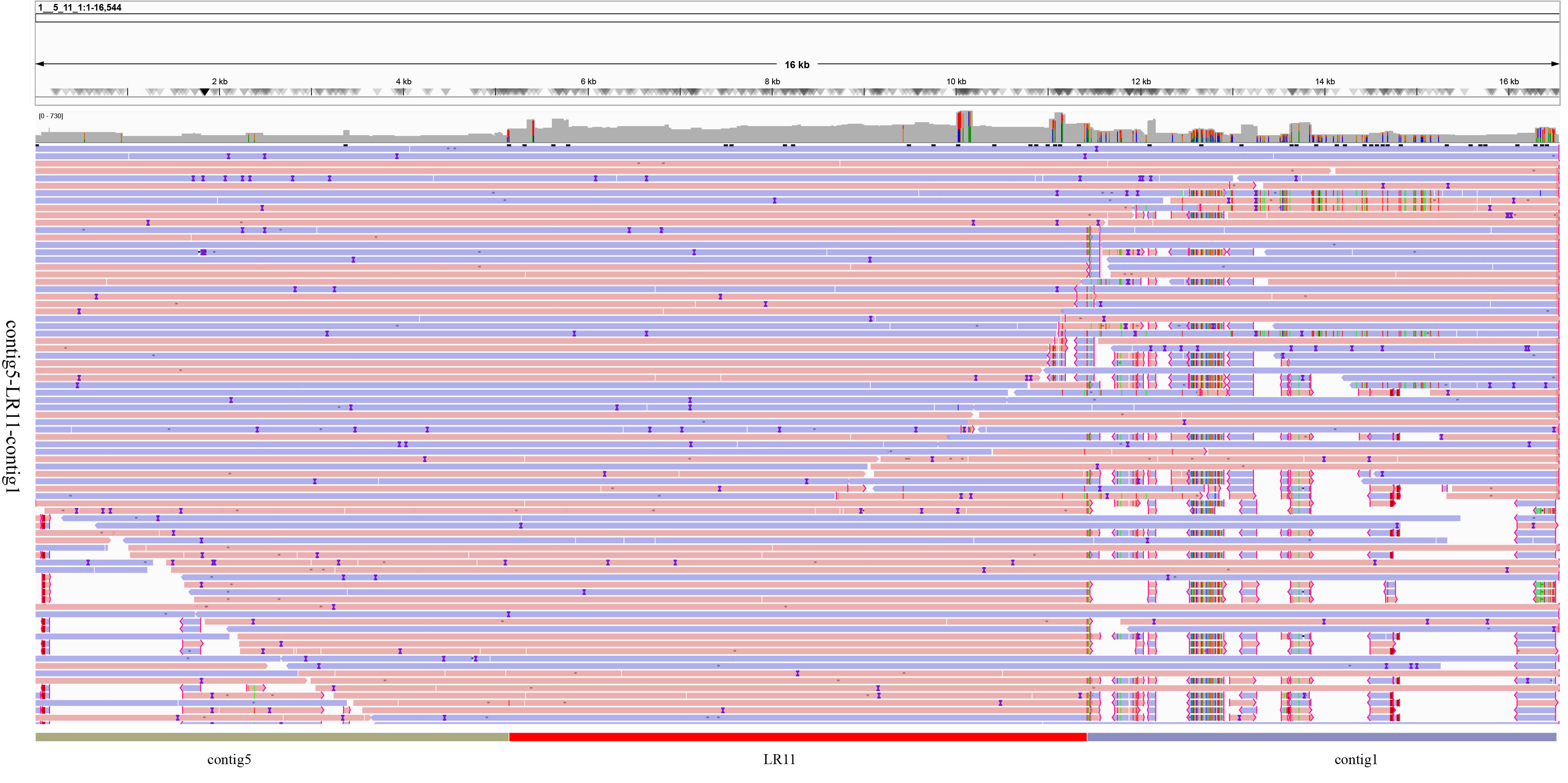


**Supplementary Figure S9.** The mapping results of the regions including the contig5-LR11-contig1 sequences in *C. carlesii* mitogenome.


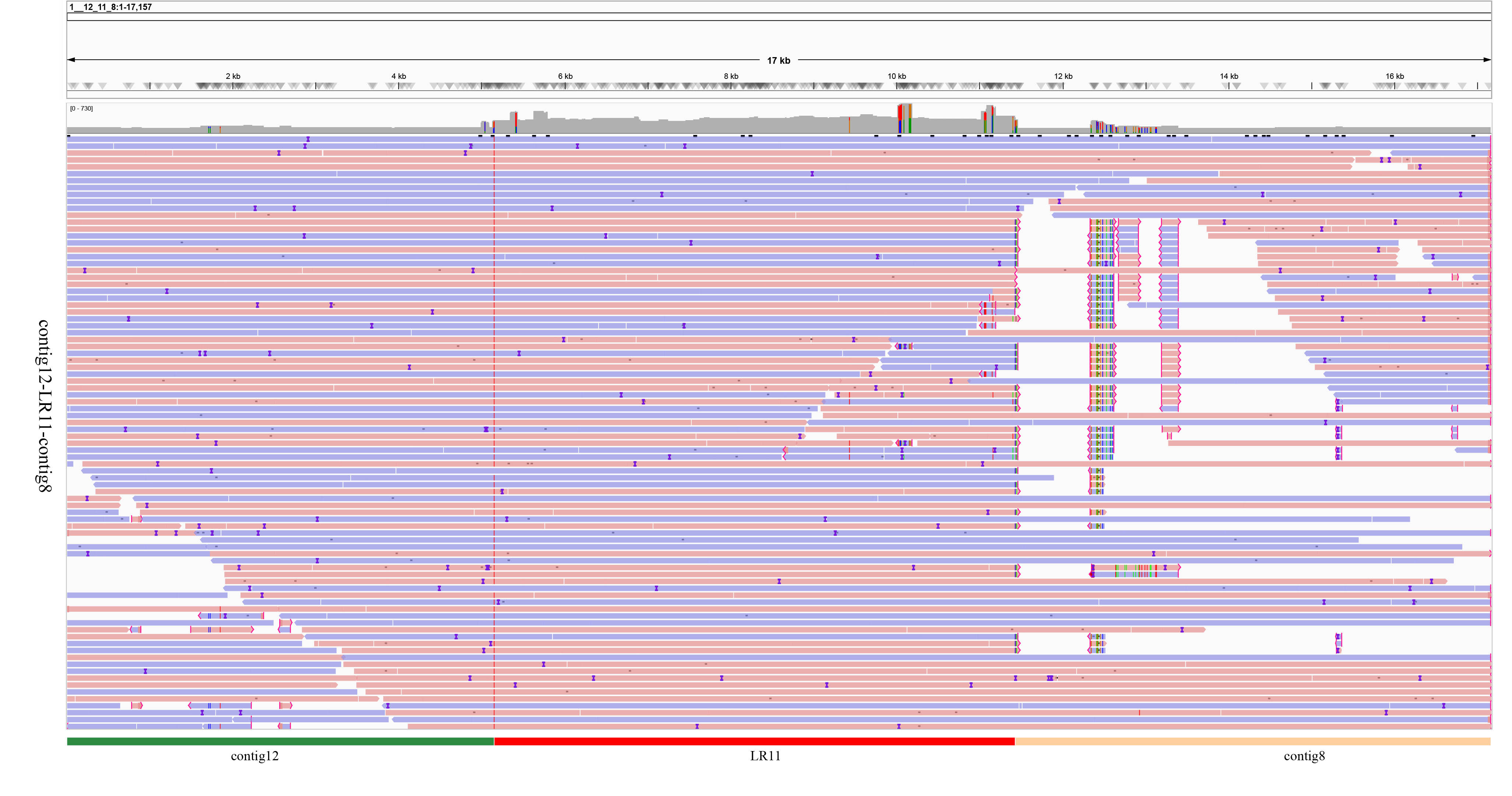


**Supplementary Figure S10.** The mapping results of the regions including the contig12-LR11-contig8 sequences in *C. carlesii* mitogenome.


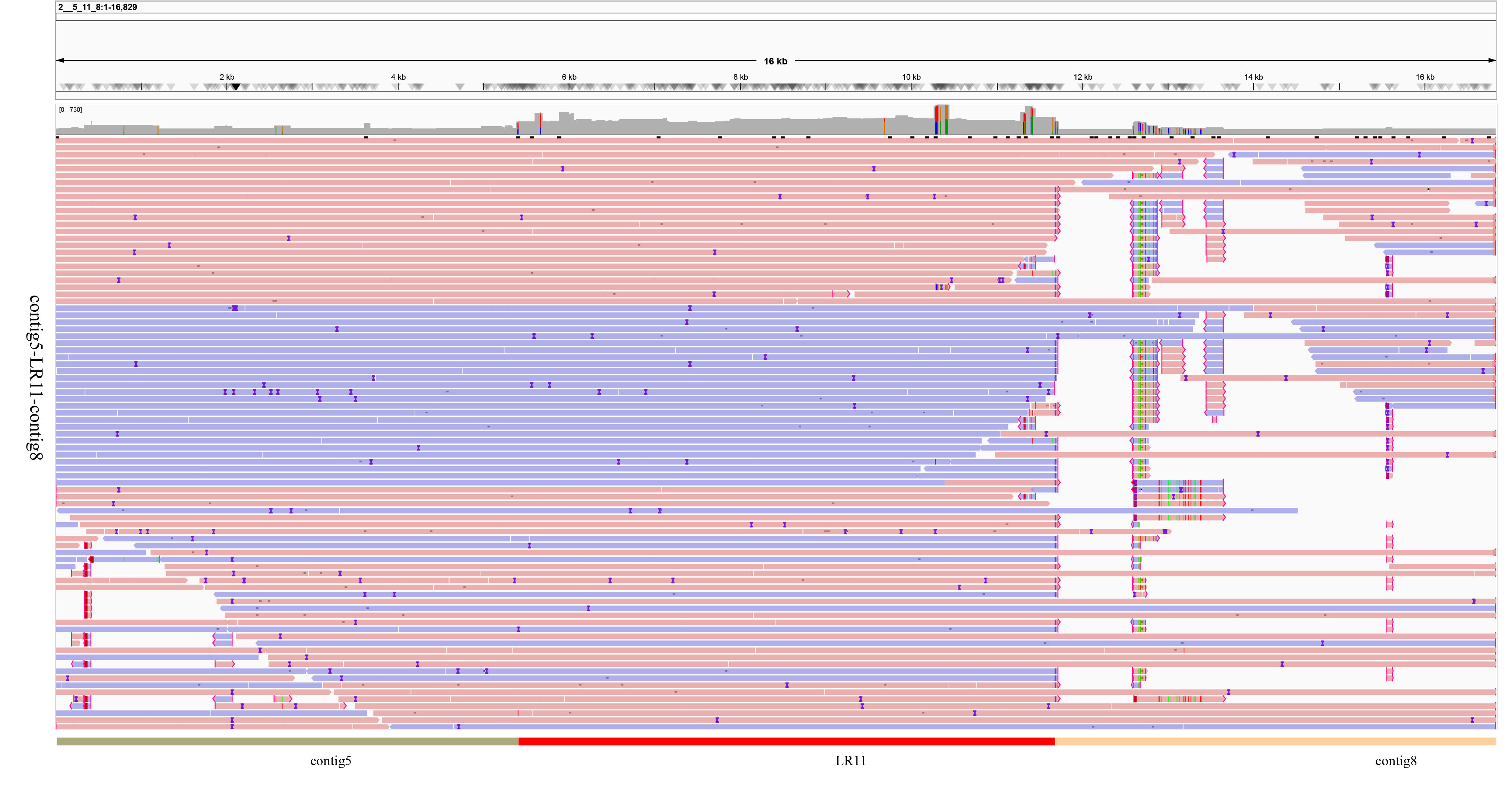


**Supplementary Figure S11.** The mapping results of the regions including the contig5-LR11-contig8 sequences in *C. carlesii* mitogenome.


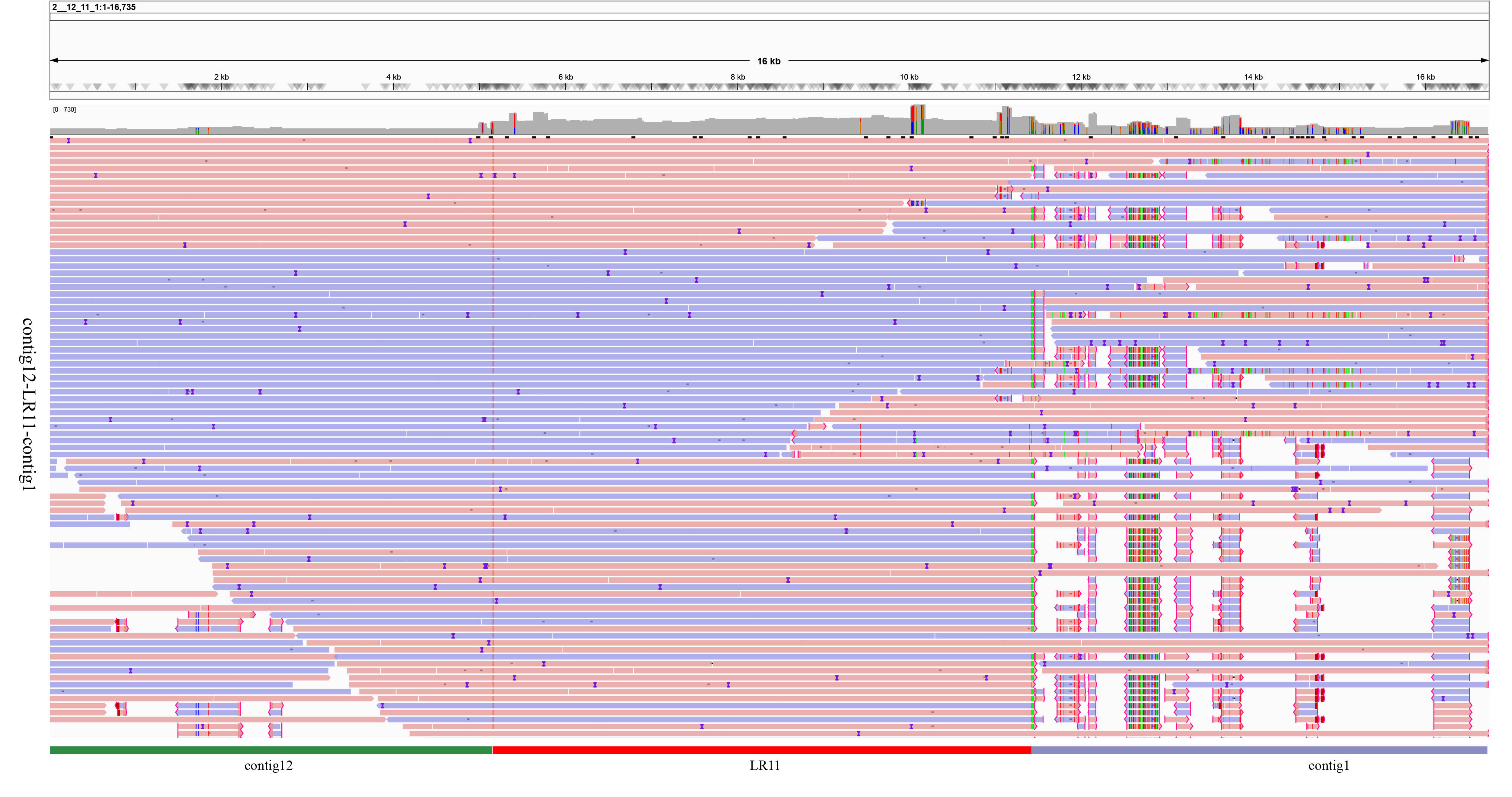


**Supplementary Figure S12.** The mapping results of the regions including the contig12-LR11-contig1 sequences in *C. carlesii* mitogenome.


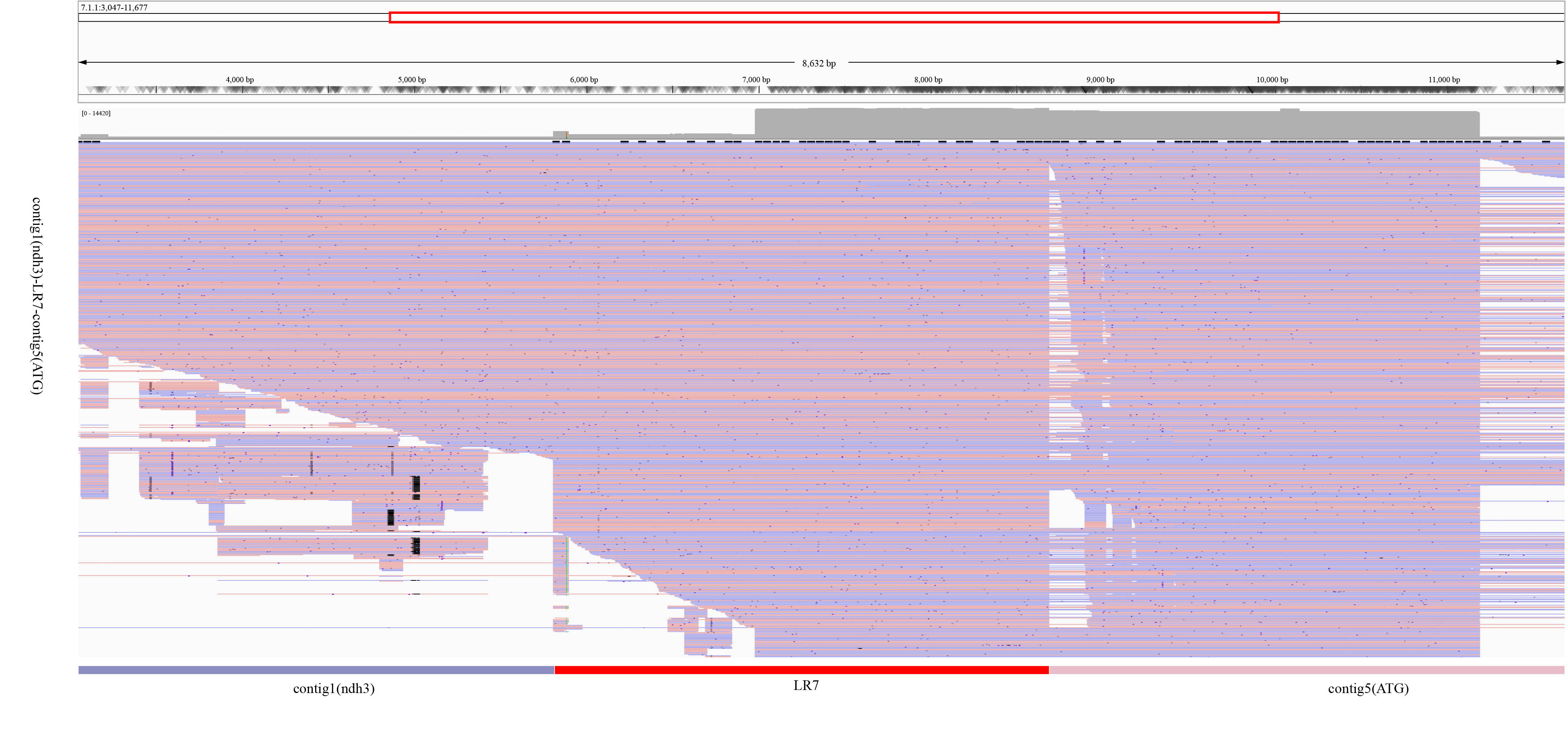


**Supplementary Figure S13.** The mapping results of the regions including the contig1-LR7-contig5 sequences in *Ca. henryi* mitogenome.


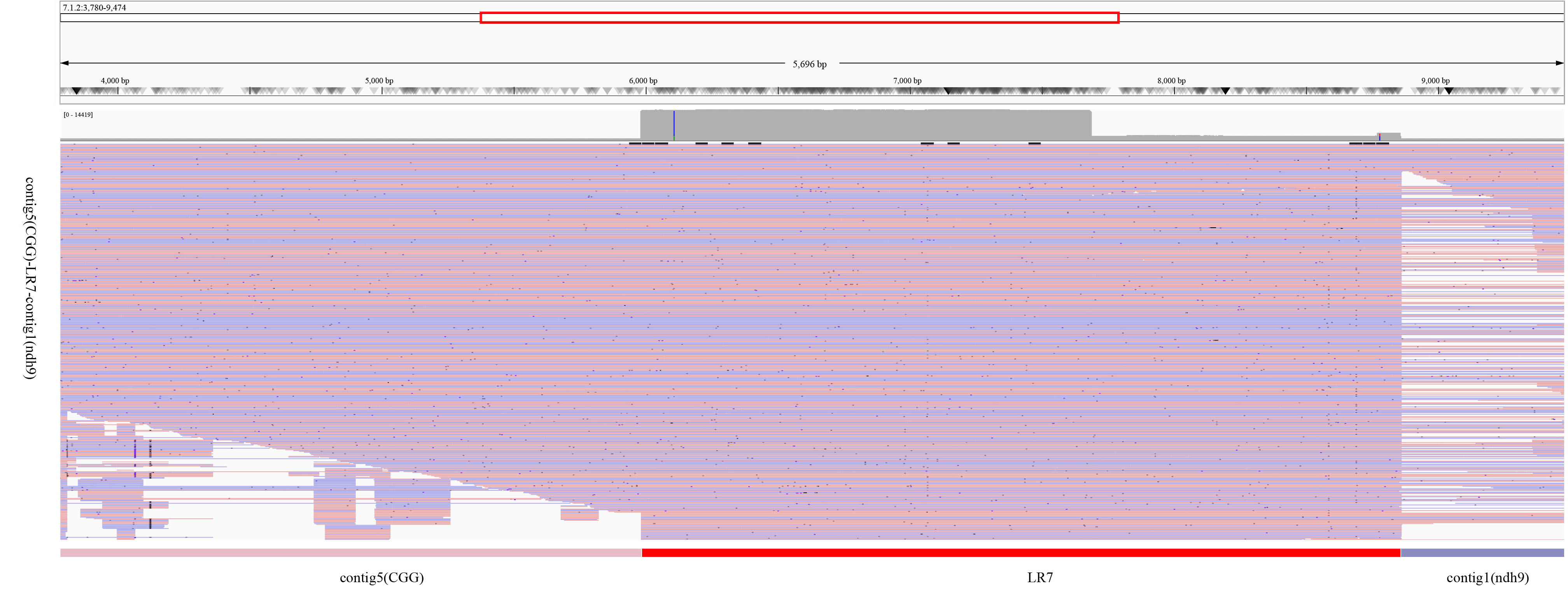


**Supplementary Figure S14.** The mapping results of the regions including the contig5-LR7-contig1 sequences in *Ca. henryi* mitogenome.


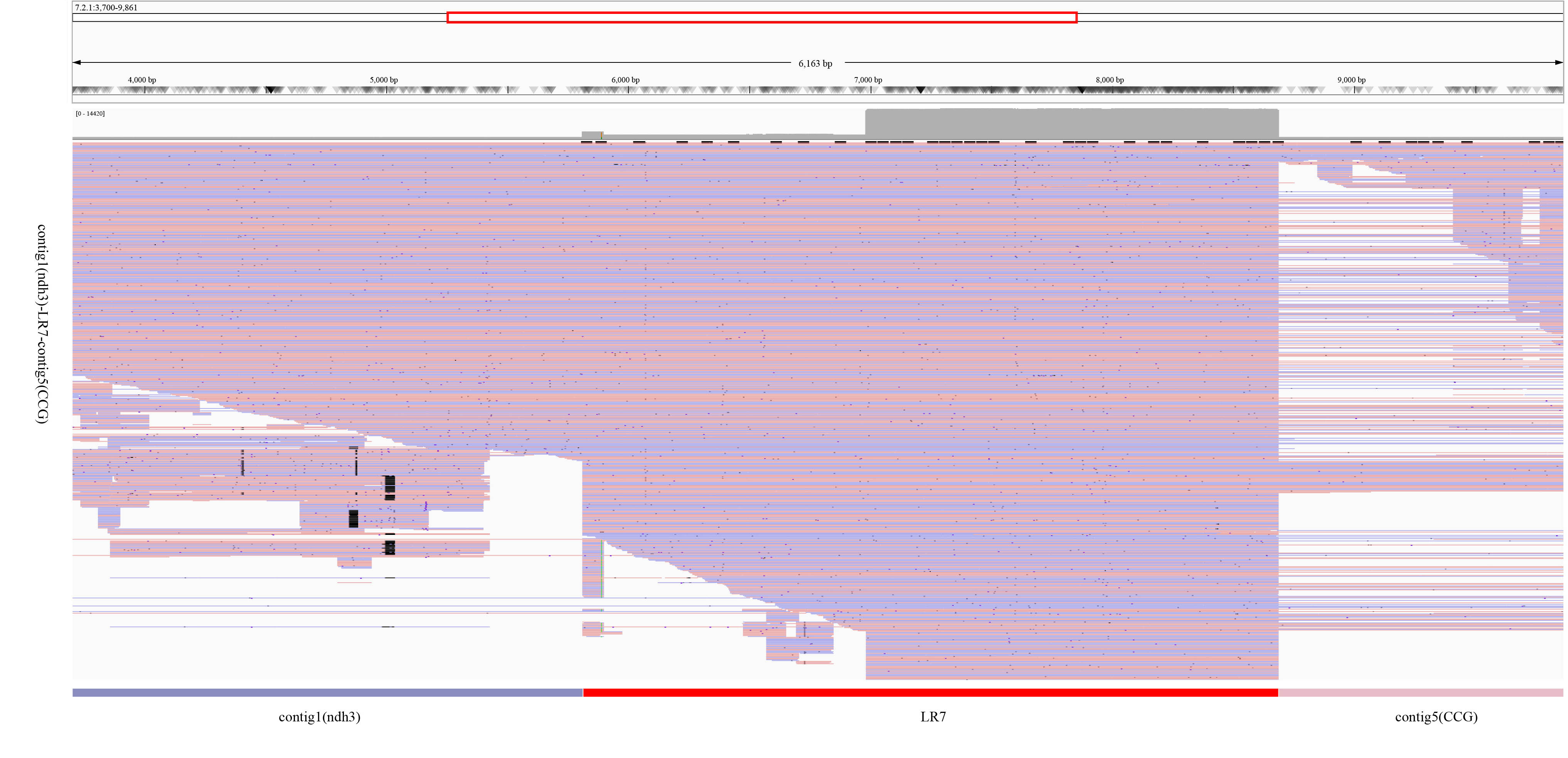


**Supplementary Figure S15.** The mapping results of the regions including the contig1-LR7-contig5 sequences in *Ca. henryi* mitogenome.


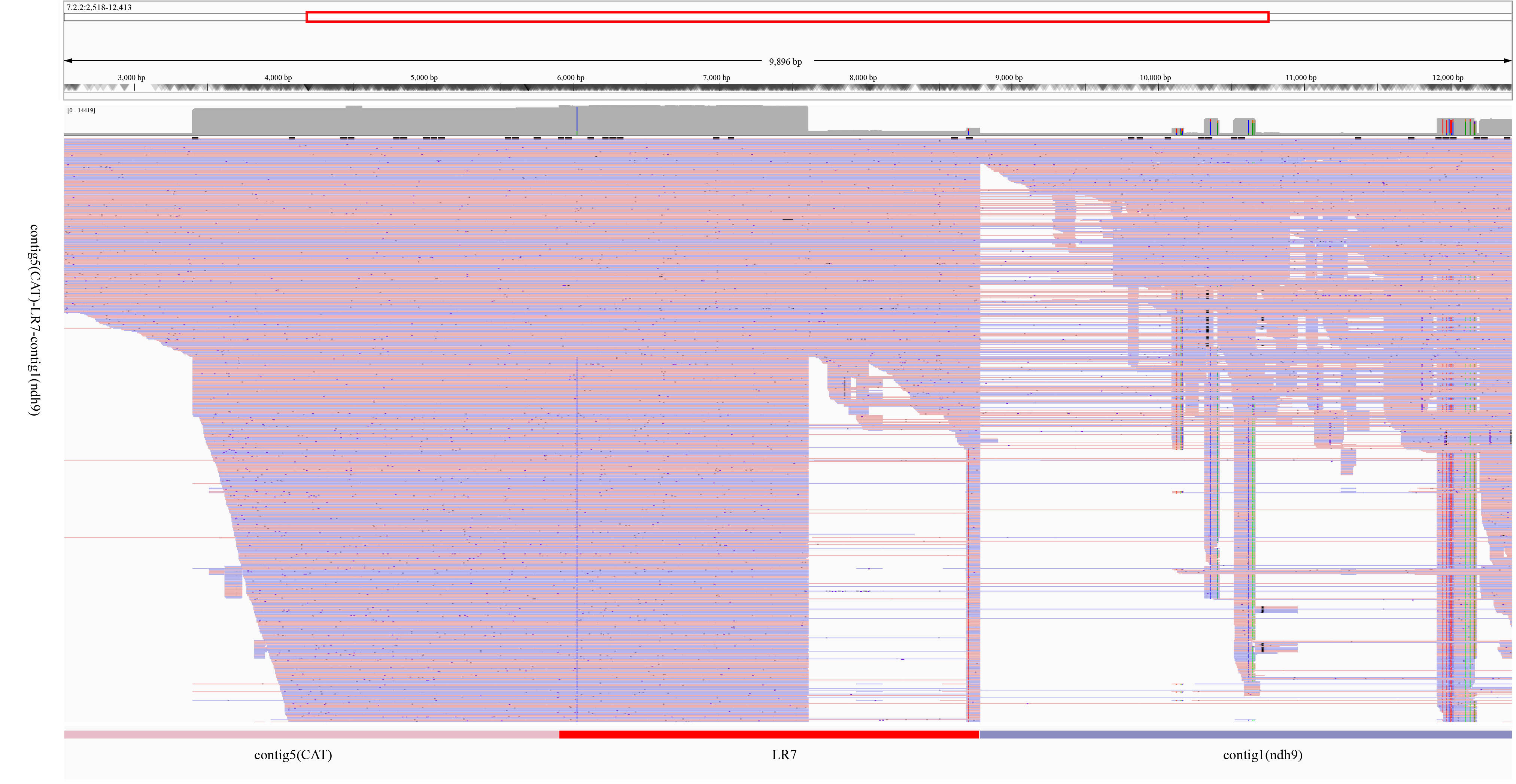


**Supplementary Figure S16.** The mapping results of the regions including the contig5-LR7-contig1 sequences in *Ca. henryi* mitogenome.


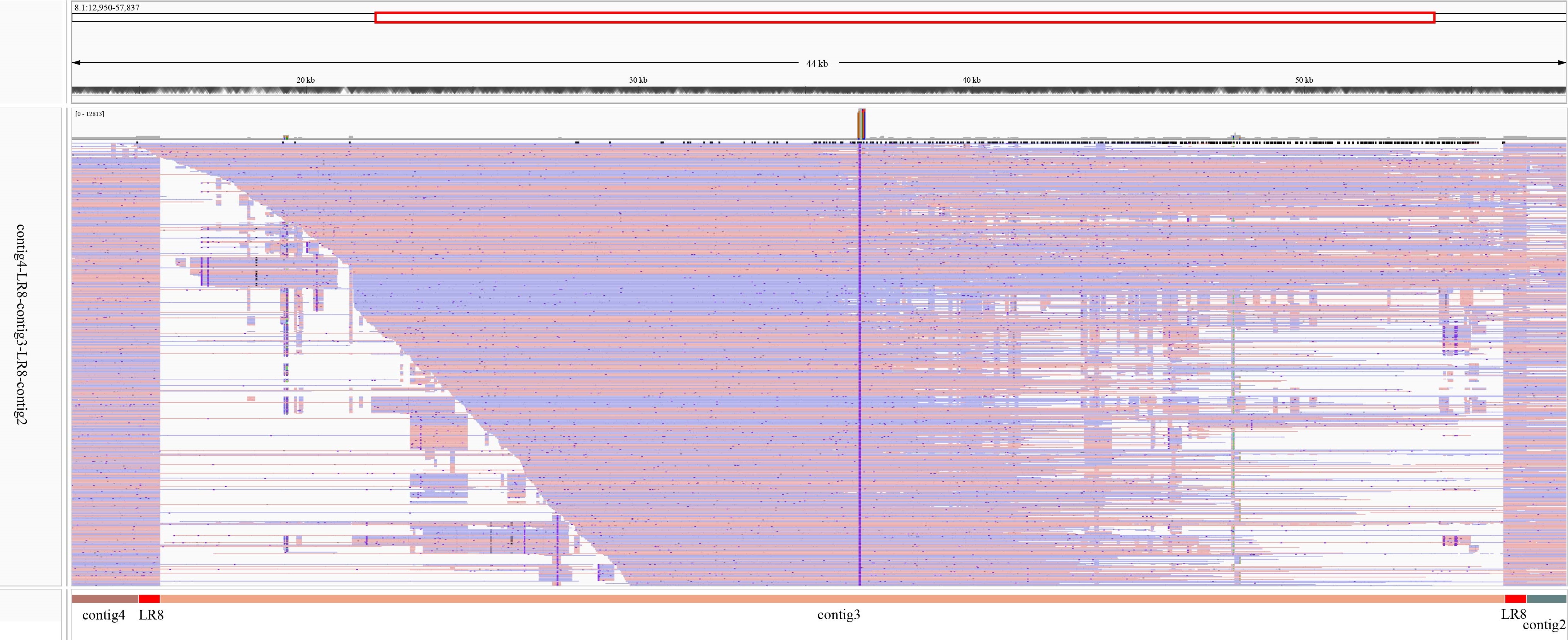


**Supplementary Figure S17.** The mapping results of the regions including the contig4-LR8-contig3 and contig3-LR8-contig2 sequences in *Ca. henryi* mitogenome.


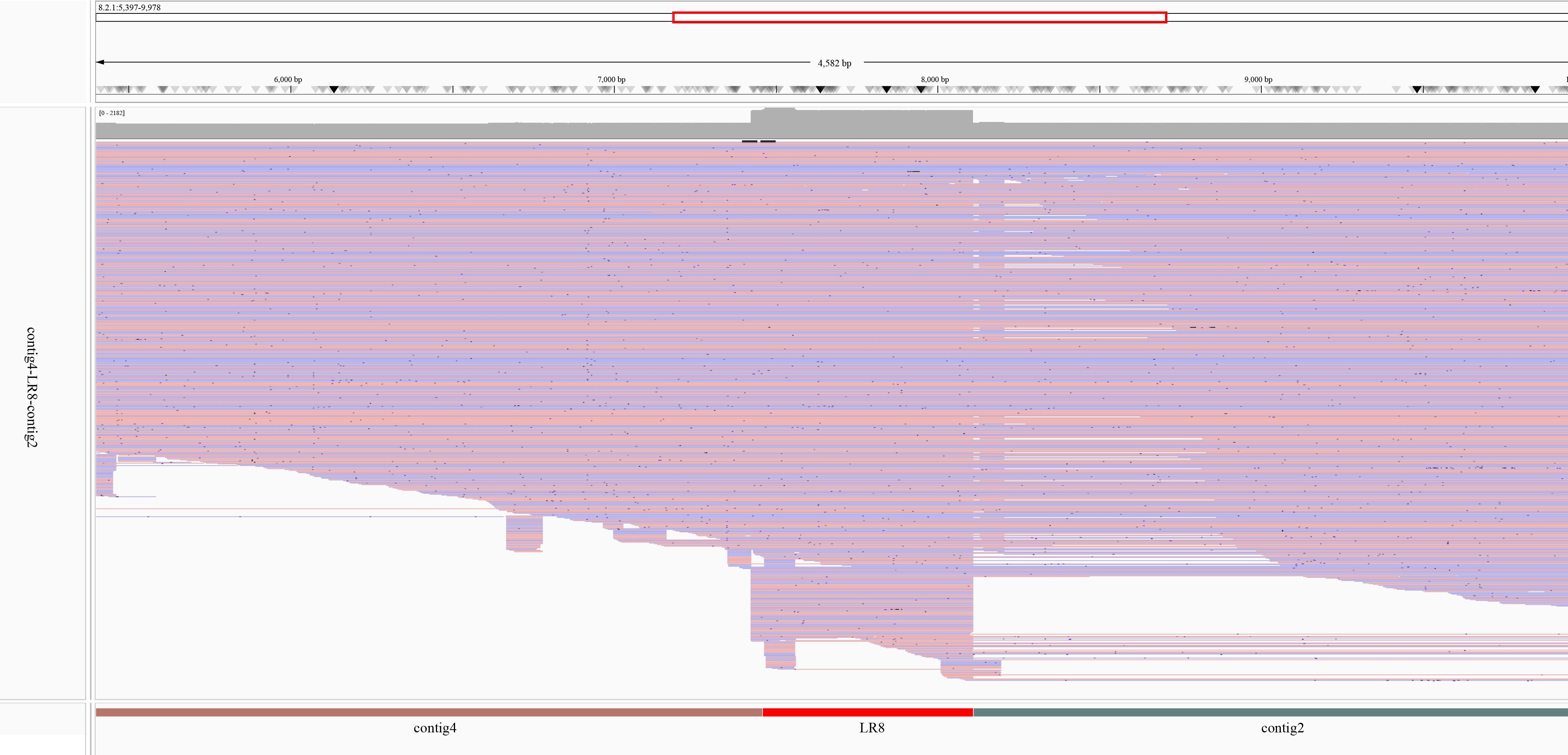


**Supplementary Figure S18.** The mapping results of the regions including the contig4-LR8-contig2 sequences in *Ca. henryi* mitogenome.


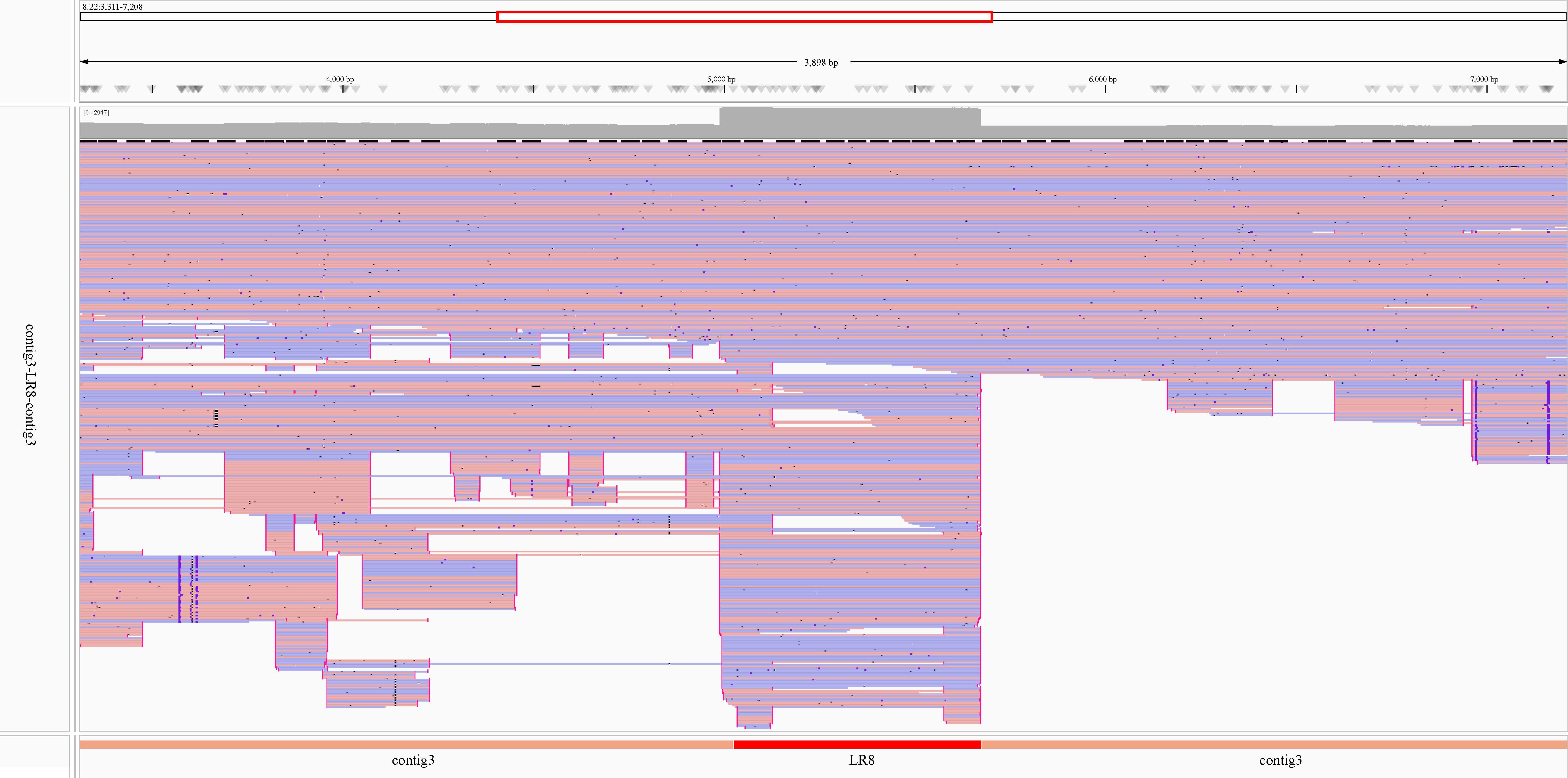


**Supplementary Figure S19.** The mapping results of the regions including the contig3-LR8-contig3 sequences in *Ca. henryi* mitogenome.


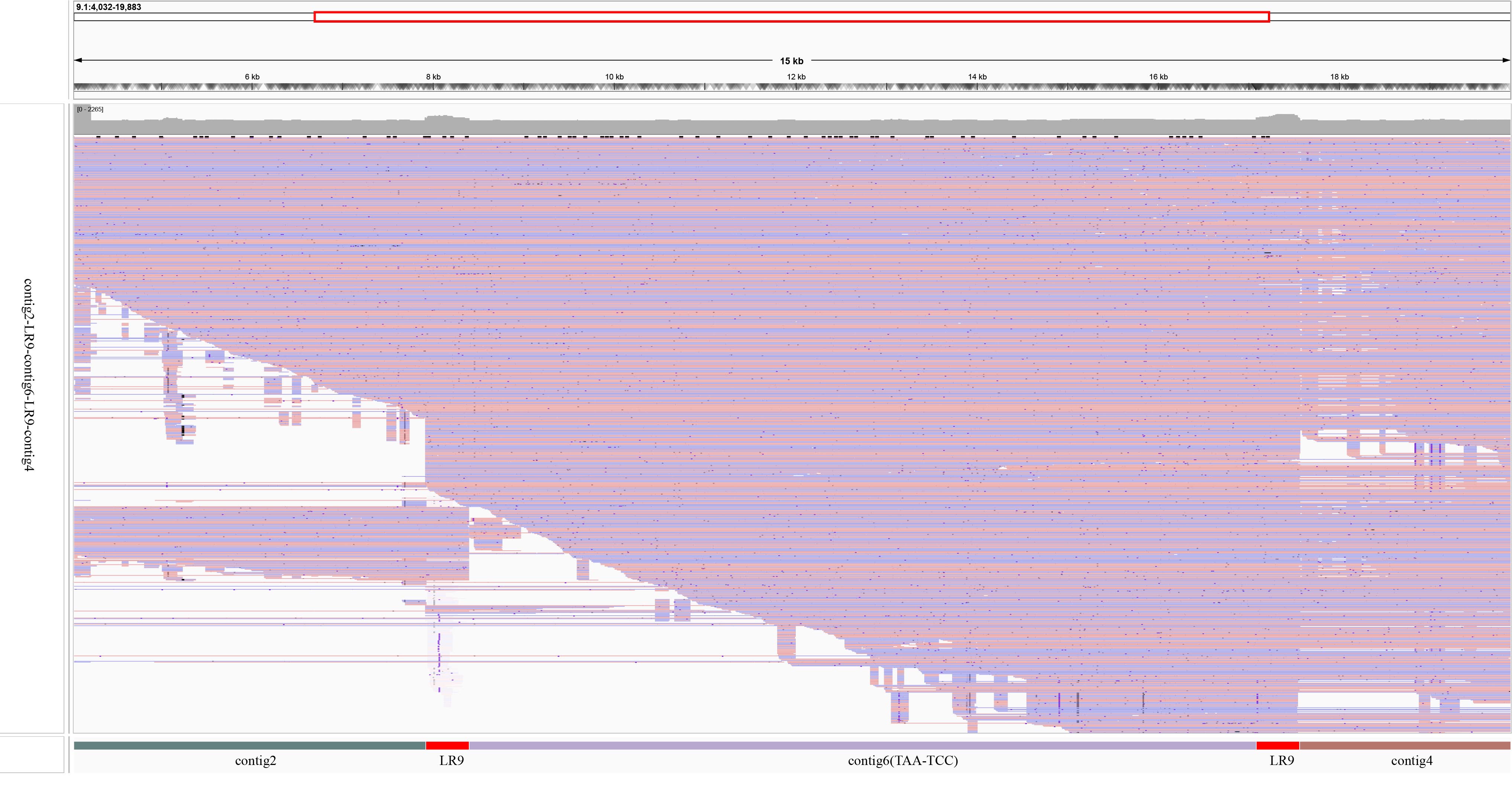


**Supplementary Figure S20.** The mapping results of the regions including the contig2-LR9-contig6 and contig6-LR9-contig4 sequences in *Ca. henryi* mitogenome.


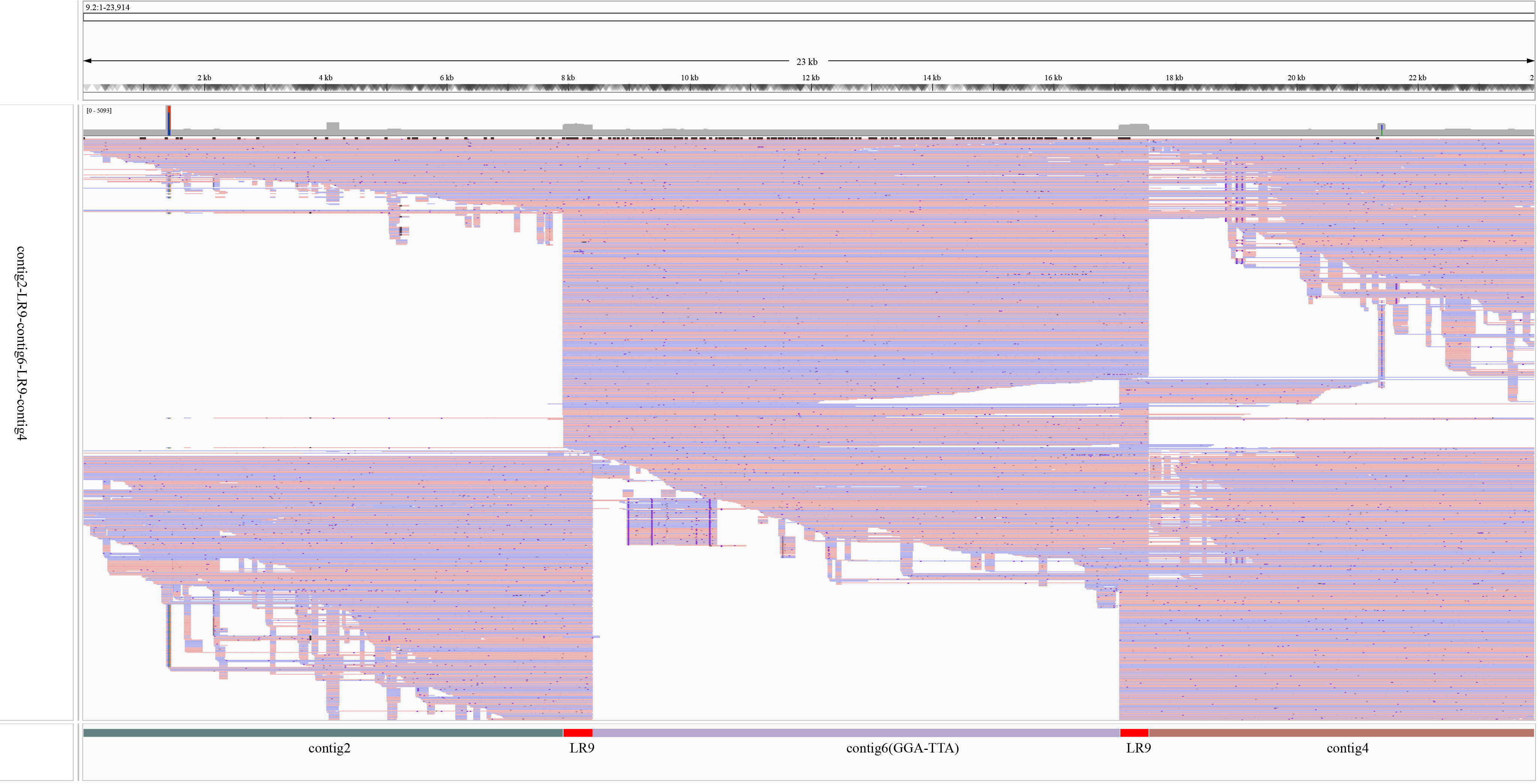


**Supplementary Figure S21.** The mapping results of the regions including the contig2-LR9-contig6 and contig6-LR9-contig4 sequences in *Ca. henryi* mitogenome.


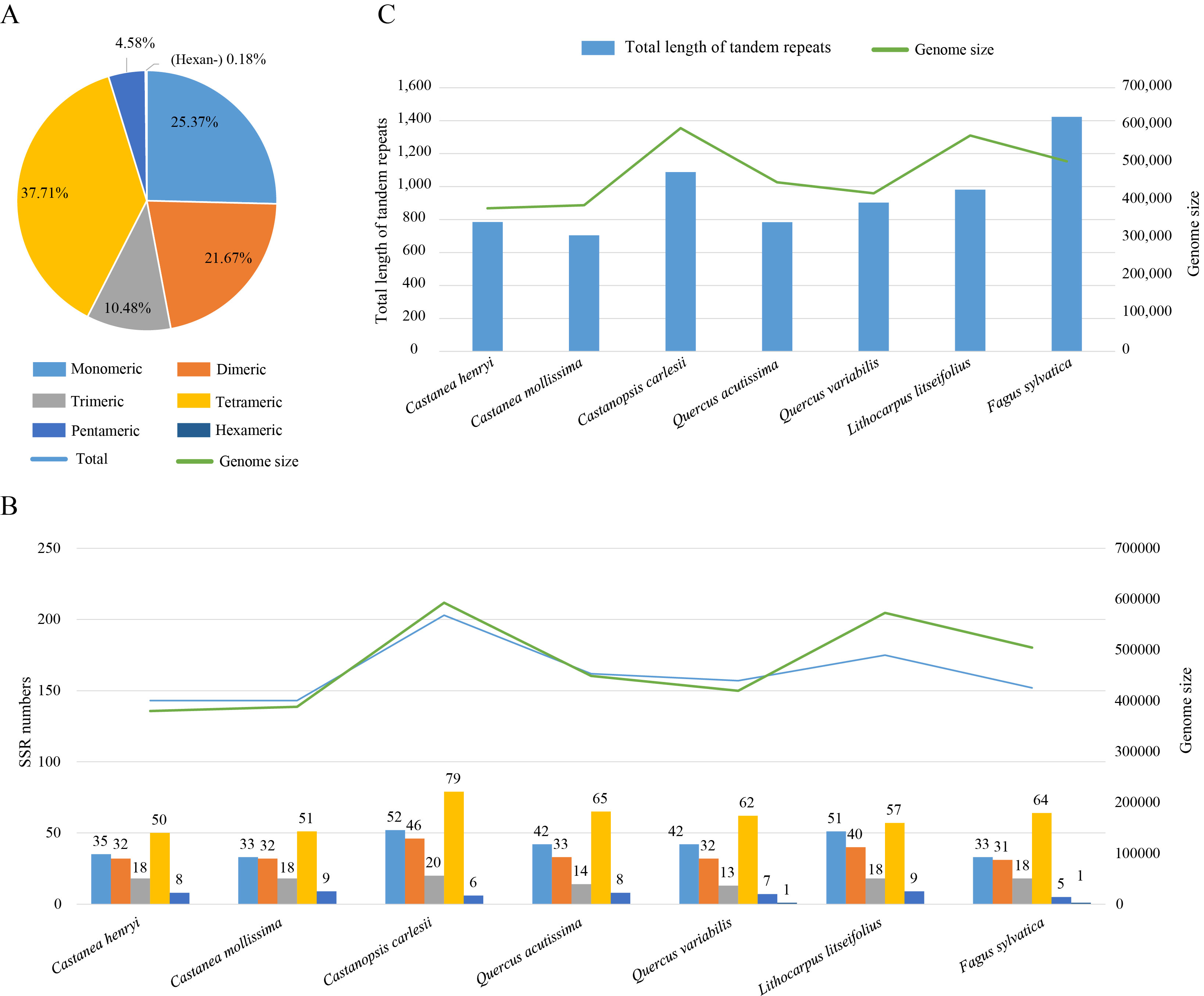


**Supplementary Figure S22.** Analyses of SSR and tandem repeats among seven Fagaceae mitogenomes. (A) Percentage of SSR types. (B) Number of SSRs and their types. (C) Number of tandem repeats.


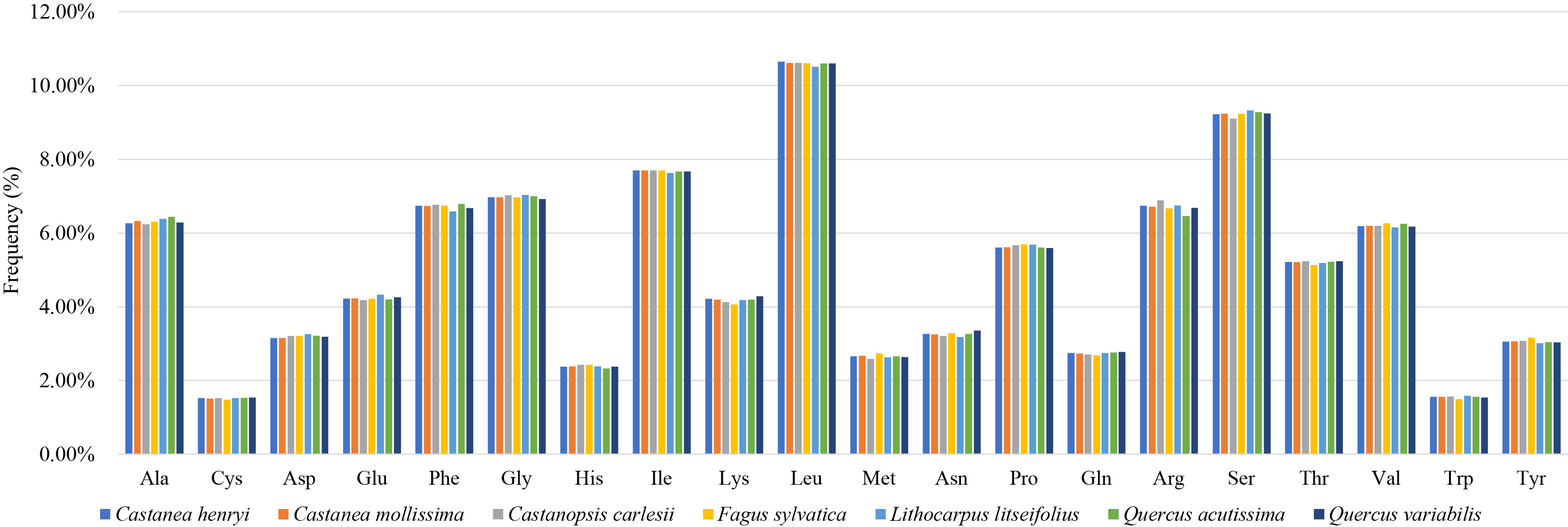


**Supplementary Figure S23.** Amino acid frequencies among seven Fagaceae mitogenomes PCGs.


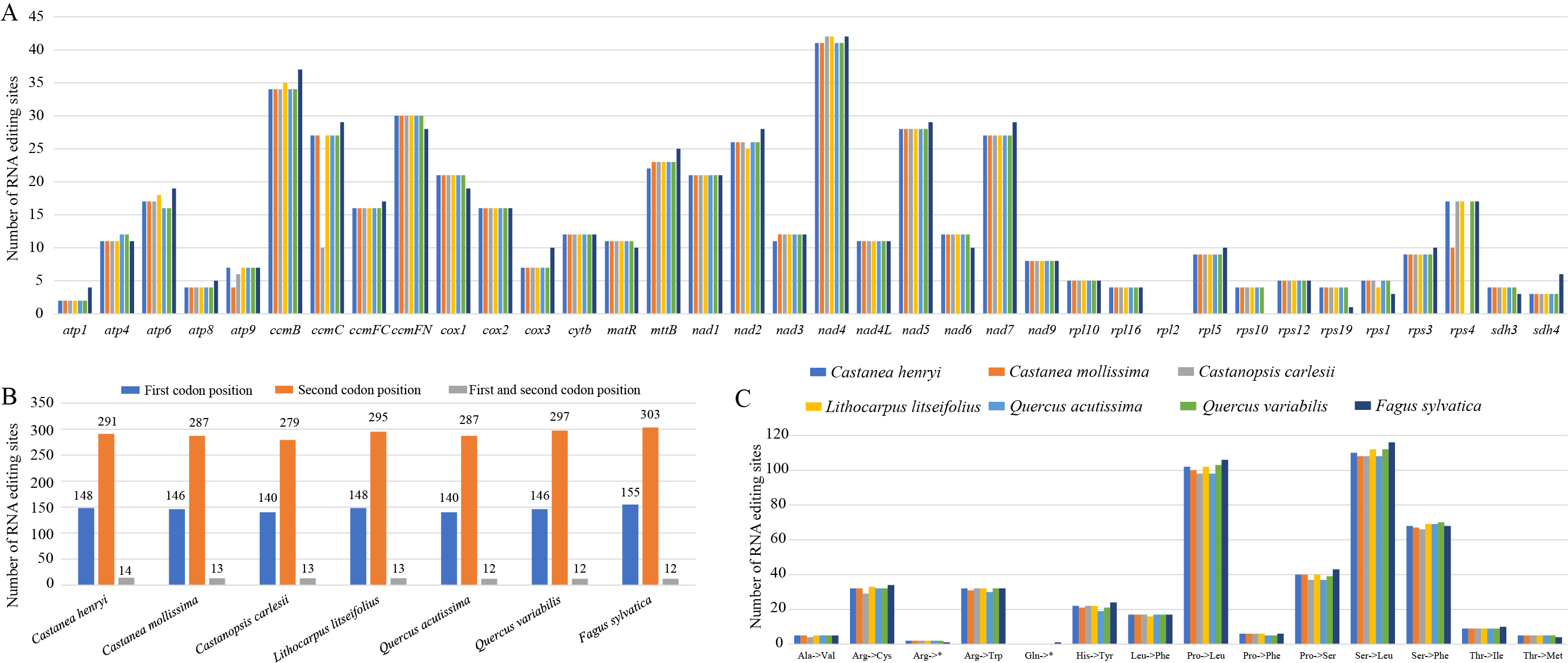


**Supplementary Figure S24.** Comparison of RNA editing sites among seven Fagaceae mitogenomes PCGs. (A) Number of RNA editing sites at different PCGs. (B) Number of RNA editing sites at the first, second, and first&second codon positions. (C) Number of RNA editing sites causing different amino acid conversion.
